# Buzzing with Intelligence: A Systematic Review of Smart Beehive Technologies

**DOI:** 10.3390/s25175359

**Published:** 2025-08-29

**Authors:** Josip Šabić, Toni Perković, Petar Šolić, Ljiljana Šerić

**Affiliations:** Faculty of Electrical Engineering, Mechanical Engineering and Naval Architecture in Split, University of Split, 21000 Split, Croatia; psolic@fesb.hr (P.Š.); ljiljana@fesb.hr (L.Š.)

**Keywords:** smart beehives, precision apiculture, hive monitoring, intelligent systems, Internet of Things, datasets

## Abstract

Smart-beehive technologies represent a paradigm shift in beekeeping, transitioning from traditional, reactive methods toward proactive, data-driven management. This systematic literature review investigates the current landscape of intelligent systems applied to beehives, focusing on the integration of IoT-based monitoring, sensor modalities, machine learning techniques, and their applications in precision apiculture. The review adheres to PRISMA guidelines and analyzes 135 peer-reviewed publications identified through searches of Web of Science, IEEE Xplore, and Scopus between 1990 and 2025. It addresses key research questions related to the role of intelligent systems in early problem detection, hive condition monitoring, and predictive intervention. Common sensor types include environmental, acoustic, visual, and structural modalities, each supporting diverse functional goals such as health assessment, behavior analysis, and forecasting. A notable trend toward deep learning, computer vision, and multimodal sensor fusion is evident, particularly in applications involving disease detection and colony behavior modeling. Furthermore, the review highlights a growing corpus of publicly available datasets critical for the training and evaluation of machine learning models. Despite the promising developments, challenges remain in system integration, dataset standardization, and large-scale deployment. This review offers a comprehensive foundation for the advancement of smart apiculture technologies, aiming to improve colony health, productivity, and resilience in increasingly complex environmental conditions.

## 1. Introduction

Honeybees (*Apis mellifera*) are essential pollinators in ecosystems and agricultural systems worldwide. However, their populations have been declining due to multiple stressors including climate change, pesticide exposure, habitat loss, and pathogens [1,2,3]. This decline threatens global food security and biodiversity and has motivated researchers to develop more efficient and non-invasive hive monitoring strategies [4,5].

Precision apiculture, or smart beekeeping, uses embedded sensors, wireless communications, and AI algorithms to monitor hives in real-time and support timely interventions [6,7,8]. These systems can capture acoustic signals, environmental conditions, hive weight, and visual cues to detect anomalies such as swarming, queen loss, or disease presence [9,10,11].

Technological advancements include the integration of wireless sensor networks (WSNs) and low-power communication protocols such as ZigBee, LoRa, and NB-IoT [12,13]. In parallel, artificial intelligence techniques, including convolutional neural networks (CNNs), support vector machines (SVMs), and random forests, are increasingly used to analyze hive soundscapes, image data, and temporal patterns [14,15,16].

Despite the potential of these systems, the literature remains fragmented. Most studies focus on proof-of-concept deployments with limited duration and controlled environments, lacking robustness and generalizability [2,8]. Moreover, there is limited cross-comparison of sensing approaches and a lack of publicly available datasets for benchmarking [17,18]. These issues persist despite growing interest and publication volume: heterogeneous application goals, diverse sensing modalities, and a variety of communication protocols and data analysis methods all hinder the synthesis of best practices and limit cross-study generalizability. Many systems are still prototypes that have not been validated over long time periods or in real-world apiaries, making it difficult to assess their robustness [19,20].

To address these gaps, this systematic literature review analyzes 135 peer-reviewed publications between 1990 and 2025. We categorize the reviewed works across six dimensions: sensor modality, communication method, data storage approach, data processing algorithm, and application objective, which collectively serve to inform the sixth dimension—hive state classification. To ensure transparency and reproducibility, this systematic review follows the PRISMA (Preferred Reporting Items for Systematic Reviews and Meta-Analyses) guidelines. PRISMA provides a structured framework for conducting and reporting systematic reviews.The aim of this systematic review is to answer the following research questions (RQ):

- **RQ1:** What types of sensing modalities are most commonly used in smart beehive systems?
- **RQ2:** In which application domains are smart technologies for beehives being deployed, and how have these focal areas evolved over time?
- **RQ3:** Which data analysis and machine learning methods have been applied, and how prevalent are advanced techniques in comparison to classical approaches?
- **RQ4:** What technical and practical limitations are reported across these studies?
- **RQ5:** What publicly available datasets exist for smart-beehive research, what data modalities do they include, and how are these datasets labeled and used to develop or evaluate machine learning models?

Through this analysis, we identify prevailing trends, methodological limitations, and areas for future research in smart apiculture systems.

## 2. Materials and Methods

This systematic review was conducted in accordance with the PRISMA 2020 guidelines [21], including development of a review protocol, a comprehensive multi-database search, and documentation of the screening process in a PRISMA flow diagram in Section 3.

### 2.1. Eligibility Criteria

The primary eligibility criteria for inclusion in this systematic literature review were defined to identify studies that implement intelligent systems within the scope of precision apiculture, particularly involving smart beehive technologies. The broader term ’smart beehive’ was utilized to encompass studies that apply various smart technologies such as sensors, IoT (Internet of Things), artificial intelligence (AI), machine learning (ML), and data analytics specifically targeted at beekeeping and hive management. To enhance study quality, we further restricted the corpus to peer-reviewed publications reporting empirical data and excluded editorials, abstracts, theses, and articles lacking reproducible methods or data. Two authors independently screened titles and abstracts and assessed full texts against these criteria; disagreements were resolved by discussion or, when necessary, by consulting a third reviewer. In addition, a rudimentary quality assessment was applied during full-text screening: we required each study to describe its sensor configuration, provide at least one quantitative metric (e.g., accuracy, precision, recall or error) and report experimental conditions (sample size, location or duration). Papers lacking these methodological details were excluded to ensure reproducibility of the extracted information.

Studies that focused solely on traditional beekeeping practices without technological integration, or those dealing only with biological or ecological aspects without applying sensor technology or data-driven analytical methods, were excluded.

Only peer-reviewed articles and conference papers published in English between January 1990 and April 2025 were considered. The detailed eligibility criteria utilized for this systematic review are summarized in Table 1.

### 2.2. Information Sources

A search of the Web of Science, IEEE Xplore, and Scopus databases was conducted on 7 April 2025, to identify relevant scientific publications related to intelligent systems in beekeeping. These databases were chosen for their broad coverage of peer-reviewed literature in engineering, agriculture, and computer science. The search included all publications available up to 7 April 2025.

### 2.3. Search Strategy

A comprehensive literature search was conducted on 7 April 2025, using three major scientific databases: Web of Science, IEEE Xplore, and Scopus. These databases were selected for their extensive coverage of peer-reviewed scientific and engineering literature relevant to intelligent systems and applied technologies. The search strategy was developed collaboratively by two reviewers and was piloted and iteratively refined to balance recall and specificity.

The search strategy was carefully adapted to the syntax and filtering capabilities of each database, with a temporal range covering publications from 1990 to 2025. In all cases, we combined general technology keywords (precision, smart, intelligent, automated) with beekeeping-specific terms (beekeeping, beehive, apiculture, apiary) using Boolean operators. Additional phrase searches (“precision beekeeping”, “smart beehive”) were included to capture variations. The aim was to identify studies focused on the application of intelligent systems in beekeeping, particularly involving smart beehive technologies. In the Web of Science database, the search query combined general terms such as precision, smart, intelligent, and automated with domain-specific keywords like beekeeping, beehive, apiculture, and apiary. These were searched within all fields using the Boolean operator OR to ensure inclusivity. Filters were applied to restrict the document type to journal articles and conference proceedings, and the date range was set from 1990 to 2025.

For IEEE Xplore, the query was adapted to search across all metadata fields using the same combinations of general and domain-specific terms. The results were further refined to include only journal and conference publications. Across all databases we restricted the language to English and excluded document types such as editorial notes, letters and abstracts. Where available, we applied citation indexing filters to prioritize peer-reviewed literature.

In Scopus, the search was conducted within titles, abstracts, and keywords using an equivalent Boolean logic structure. Publications were filtered to include only articles and conference papers written in English and published after 1990.

An overview of the search queries applied to each database, including Boolean logic and filtering criteria, is provided in Table 2.

### 2.4. Data Extraction and Categorization

To systematically analyze and compare smart beehive systems, we conducted a structured data extraction process. Each reviewed study was annotated across six dimensions: Bibliographic Info, Sensor Type, Communication Type, Method/Technique Type, Goal Category, and Key Aspects. These dimensions enable uniform representation of heterogeneous systems and serve as the foundation for subsequent visualizations and synthesis.

**Figure 1:**
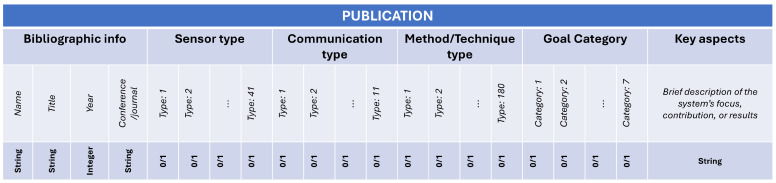
Structure of the data extraction matrix used to encode publications.

Figure 1 illustrates the structure of the extraction matrix. For each publication, binary encoding (0/1) was applied to indicate the presence of a given feature or method. Additionally, descriptive metadata was manually extracted and paraphrased from each study to provide contextual insight.

**Figure 2:**
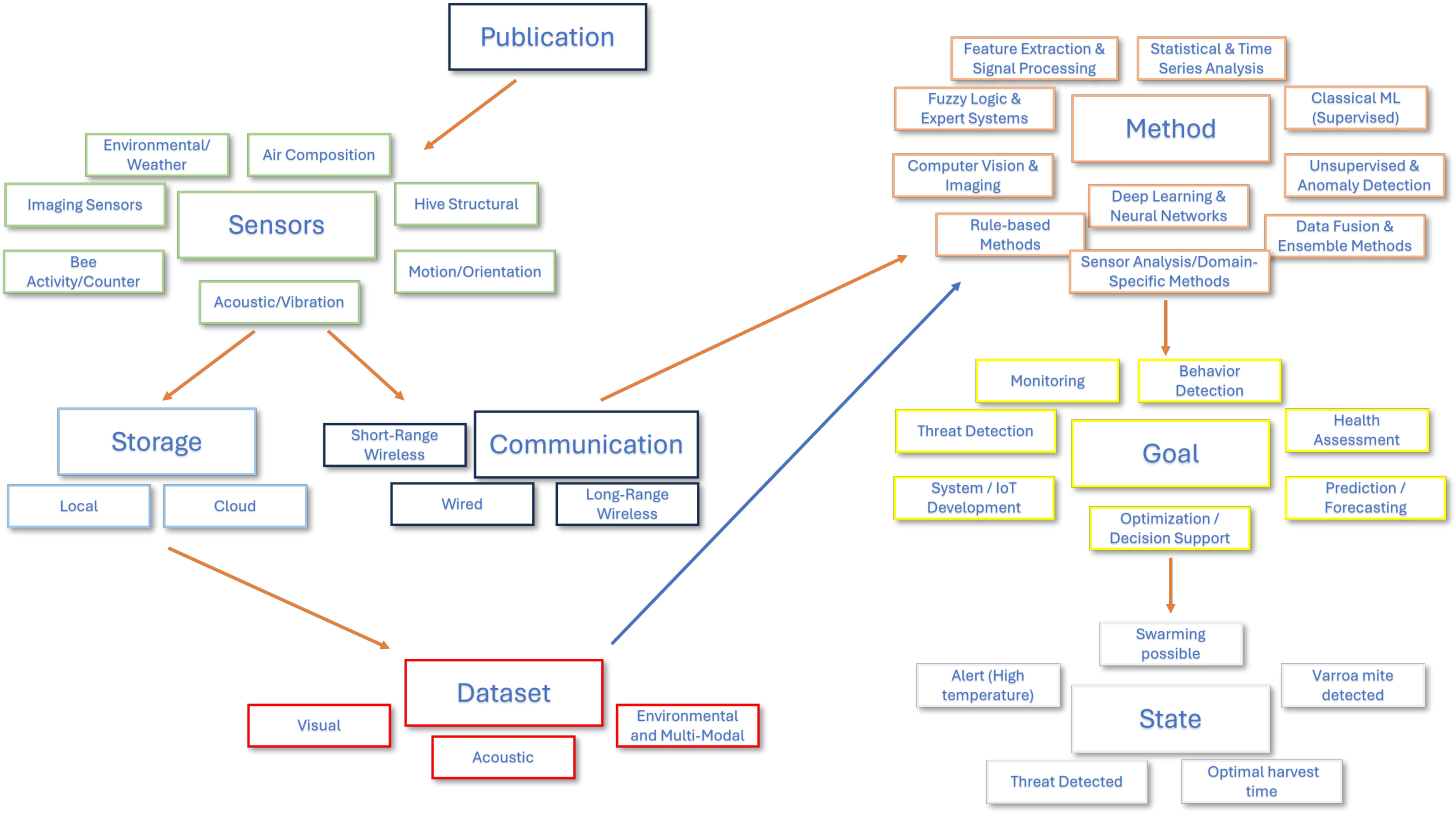
Overview of the classification taxonomy used during the data extraction process. Orange arrows indicate the typical flow starting from sensor measurements; the blue arrow denotes studies that bypass data acquisition and start from existing datasets.

To ensure clarity and reproducibility, the coding schema was supported by a formal taxonomy of categories, as shown in Figure 2. This taxonomy was used to organize the various sensor types, communication technologies, analytical methods, and goal categories. In addition to enumerating the building blocks of our review, the figure also depicts the typical flow of information through a smart-beehive system. Readings from the sensors used in a given publication or project may be logged to local or cloud storage (forming a dataset) and/or transmitted via wired, short-range or long-range wireless links to a remote computer. On the remote side, selected analytical methods are applied to these measurements in order to derive the desired goal about the hive or bee state. For completeness we note that some investigations do not collect new data but instead start from previously curated datasets—this alternative path is indicated by the blue arrow in the figure.

Each of the main categorical dimensions is described in detail below:

**Sensor Type**: This dimension captures which physical sensing technologies were used in each system. A total of 41 binary-coded sensor types were grouped into six high-level categories:

- Environmental/Weather Sensors (e.g., temperature, humidity, air pressure; these include sensors monitoring conditions both inside the hive (internal climate) and in its surroundings).
- Acoustic/Vibration Sensors (e.g., microphones, piezoelectric sensors).
- Imaging Sensors (e.g., cameras, optical counters, thermal imaging).
- Hive Structural Sensors (e.g., weight/load cells, strain gauges).
- Motion/Orientation Sensors (e.g., accelerometers, gyroscopes).
- Air Composition Sensors (e.g., CO_2_, VOC, O_2_).
- Bee Activity Counters (e.g., infrared gates, tags).

**Communication Type**: This category encodes the technologies used to transmit data from the hive. The classification includes:

- Short-Range Wireless (e.g., Zigbee, Wi-Fi, Bluetooth).
- Long-Range Wireless (e.g., LoRa, NB-IoT, Sigfox, GSM).
- Wired Communication (e.g., Ethernet, PowerLine).

**Method/Technique Type**: This is the most granular dimension, with over 180 tags, grouped into ten parent categories:

- Statistical and Time-Series Analysis (e.g., regression, correlation, ARIMA, VAR).
- Feature Extraction and Signal Processing (e.g., FFT, MFCC, DWT).
- Classical Machine Learning (e.g., SVM, Random Forest, k-NN, Naive Bayes).
- Deep Learning and Neural Networks (e.g., CNN, LSTM, Transformer-based models).
- Computer Vision and Image Analysis (e.g., contour detection, image segmentation)
- Unsupervised Learning and Anomaly Detection (e.g., clustering, outlier detection).
- Rule-Based Systems and Thresholding (e.g., thresholding (T1, T2, T3, T*), Custom swarming algorithm).
- Data Fusion and Ensemble Methods (e.g., weighted multi-criteria aggregation algorithm, Majority voting).
- Expert Systems and Fuzzy Logic (e.g., Fuzzy-stranded-NN, fuzzy logic model (FLM)).
- Sensor Analysis/Domain-Specific (e.g., BFCI formula: θ·T+b·P+c·W (weather scoring)).

**Goal Category**: Each system was also classified by its intended application, which helps contextualize the chosen sensors and methods. The following seven goal categories were used:

- Monitoring: Real-time reporting of hive metrics.
- Behavior Detection: Recognizing bee behaviors.
- Health Assessment: Detecting disease or colony vitality issues.
- Prediction/Forecasting: Estimating future events like swarming or yield.
- Optimization/Decision Support: Guiding interventions and hive management.
- System/IoT Development: Engineering and infrastructure for sensing platforms.
- Threat Detection: Identifying predators, theft, or environmental hazards.

**Key Aspects**: This field contains a brief textual summary of the study’s technical contribution, extracted from the abstract or discussion. It often includes insights into the system’s novelty, testing conditions, or dataset characteristics. Although not used for quantitative analysis, it adds interpretive richness to the dataset.

**Bibliographic Info**: For traceability, each entry includes citation metadata (author, title, year, source type) alongside classification. This enables filtering by publication date, venue type (conference/journal), or other bibliometric properties.

The result of this data extraction process is a multidimensional matrix that enables consistent, reproducible analysis across the reviewed literature. All subsequent quantitative results and visualizations—including heatmaps, frequency distributions, and co-occurrence charts—are derived from this underlying structure.

## 3. Results and Discussion

### 3.1. Corpus and Structured Summary of Included Studies

The selection of studies was conducted following the PRISMA (Preferred Reporting Items for Systematic Reviews and Meta-Analyses) 2020 guidelines. A total of 917 records were identified through database searches on 7 April 2025, across three major scientific databases: Web of Science (*n* = 409), Scopus (*n* = 377), and IEEE Xplore (*n* = 131). After automatic removal of duplicates, 532 unique records remained.

Title screening excluded 246 records based on relevance. The remaining 286 articles were subjected to abstract screening and full-text assessment, during which 151 were excluded for not meeting the inclusion criteria (e.g., lacking empirical data, not involving intelligent systems, or focusing solely on ecological aspects). Ultimately, 135 studies were retained for full analysis.

**Figure 3:**
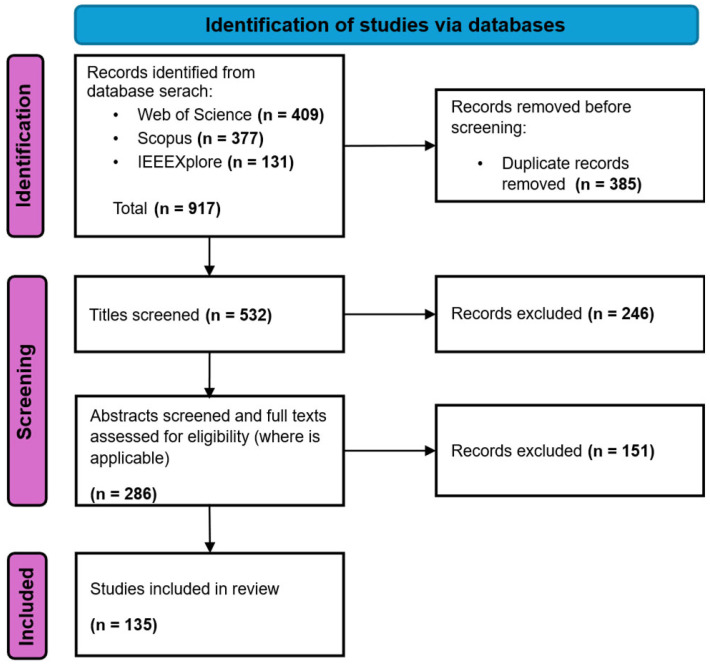
Flow diagram illustrating the publication identification and screening process following PRISMA guidelines (template adapted from Page et al. [21], CC BY 4.0).

The complete selection workflow is illustrated in Figure 3.

The selected studies were systematically coded into a structured extraction matrix, as described in Section 2.4. This matrix forms the empirical foundation for all subsequent analyses of technological patterns, analytical techniques, and system goals. A condensed version of the data is presented in Table A1 in Appendix A.

### 3.2. Overview

This organizational framework enables structured comparison of methodological approaches, sensor configurations, and system goals. By categorizing studies based on their intended purpose, the review highlights prevailing trends, emerging directions, and underexplored topics within the domain of smart beekeeping systems.

**Figure 4:**
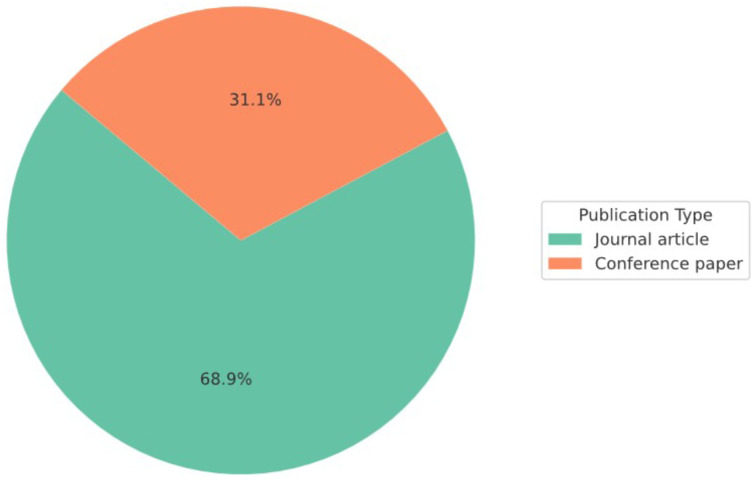
Distribution of publication types.

Figure 4 illustrates the distribution of the reviewed works by publication type and research objective. Among the 135 included studies, 93 were journal articles (68.9%) and 42 were conference papers (31.1%), indicating a preference for peer-reviewed journal publication within the research community.

**Figure 5:**
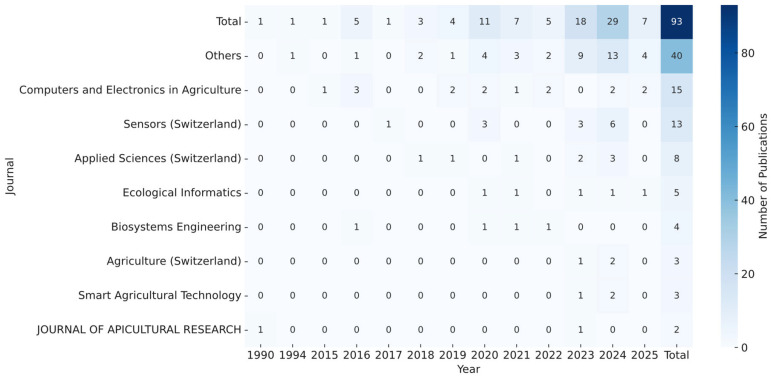
Number of reviewed publications by journal and year.

Figure 5 presents a heatmap illustrating the distribution of reviewed journal publications by year. The data reveal a steady increase in research activity related to smart beehive technologies, with a notable surge beginning around 2020. Computers and Electronics in Agriculture is the most prominent journal, publishing 15 of the reviewed studies. This reflects the strong alignment between agricultural engineering and the development of digital monitoring systems.

Other leading publication venues include Sensors, Applied Sciences, and Ecological Informatics, all of which support interdisciplinary research at the intersection of sensing technologies, environmental monitoring, and applied sciences. The “Others” category consolidates several journals with smaller contributions, indicating broader, though more dispersed, interest in the topic across additional academic platforms.

**Figure 6:**
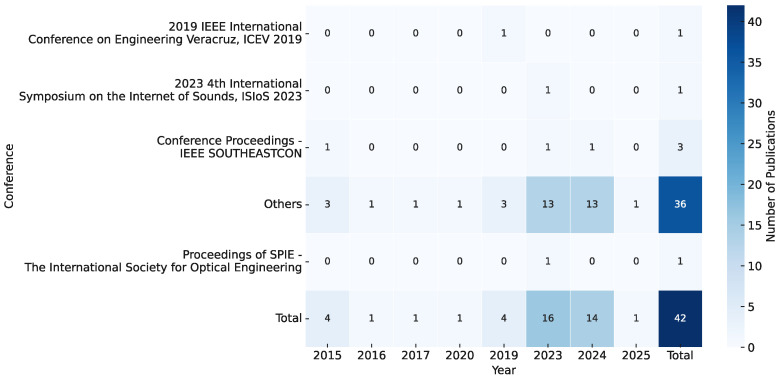
Number of reviewed publications by conference and year.

Figure 6 presents the distribution of reviewed conference publications by year. Although conferences represent a smaller share of the overall scholarly output compared to journals, they remain an essential channel for disseminating technical innovations in smart-beehive systems. Among them, IEEE SOUTHEASTCON contributes the highest number of papers, consistently serving as a venue for research on sensor systems, communication protocols, and embedded platforms relevant to apiculture.

Other conferences, including Engineering Veracruz, CSCITA, and the Internet of Sounds Symposium, each contributed a single publication, highlighting the growing interdisciplinary interest in applying domain-specific technologies to beekeeping. The “Others” category similarly comprises venues with one publication each.

Overall, the trend lines across both publication types reflect an emerging, but rapidly professionalizing research landscape. While journal publications dominate the discourse, conferences continue to provide a dynamic space for the presentation of nascent research and the cultivation of scholarly dialogue. The consistent appearance of certain venues over multiple years affirms the establishment of recurring academic communities interested in precision apiculture. Notably, our search did not retrieve contributions from major agricultural engineering conferences (e.g., ASABE or European Federation of Agricultural Engineers); this absence underscores the need for greater cross-fertilization between apiculture and mainstream agricultural technology forums. Furthermore, we observed that much of the research in this domain has been published in engineering-oriented journals and conferences, with relatively few contributions in apiculture or agriculture-specific venues. This suggests a need for better cross-disciplinary dissemination and collaboration to ensure smart beehive technologies address practical beekeeping challenges and scientific questions in apiculture.

**Figure 7:**
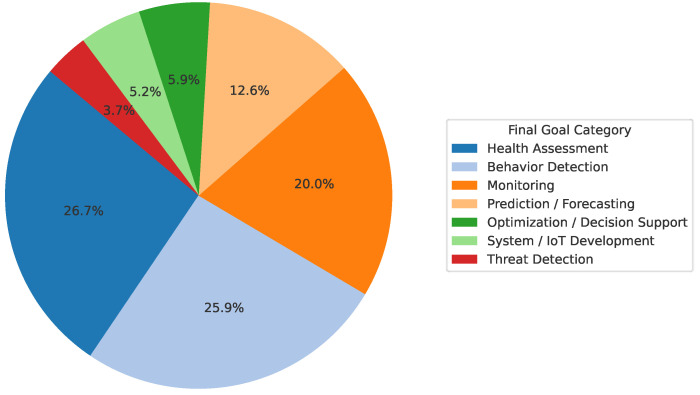
Distribution of primary research goals.

In terms of research focus, the most prevalent functional goals across the reviewed literature were Health Assessment (36 papers) and Behavior Detection (35 papers), which together comprise over half of the corpus. Health Assessment studies typically aimed to evaluate colony condition or detect signs of disease and stress, while Behavior Detection focused on identifying bee activities such as foraging, swarming, or in-hive movement patterns through visual, acoustic, or motion-based cues. The Monitoring category included 27 studies centered on real-time tracking of environmental or hive-level parameters. Prediction/Forecasting, found in 17 papers, involved anticipating future hive states such as swarming events or honey yield. Optimization/Decision Support appeared in 8 studies and focused on data-driven recommendations for hive management. System/IoT Development, present in 7 works, primarily addressed sensor integration, platform engineering, or hardware optimization. Finally, Threat Detection was the least represented category, with only 5 studies focused on identifying risks such as predators, theft, or environmental anomalies. These distributions are illustrated in Figure 7, while a complete mapping of publications by goal category is provided in Table 3.

**Figure 8:**
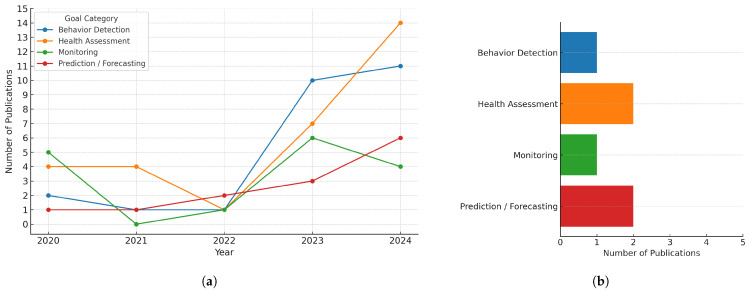
Combined visualization of publication trends for the four most common research goal categories. (**a**) shows the yearly evolution from 2020 to 2024, while (**b**) highlights current progress in 2025.

Beyond static category counts, Figure 8 illustrates the temporal progression of the four most prevalent research goals from 2020 to 2024 (Figure 8a), along with a snapshot of their current distribution in early 2025 (Figure 8b).

Health Assessment consistently remained the dominant category throughout this period, with a notable peak in 2024. This sustained trend highlights the continued importance of diagnosing colony conditions and detecting signs of disease using sensor-derived data such as audio, weight, or thermal profiles.

Behavior Detection showed a steady increase in interest, peaking in 2024 with 11 publications—making it the second most active category that year after Health Assessment. This underscores growing research attention to behavioral analysis using video, acoustic, and motion sensing, often paired with computer vision and machine learning techniques.

Monitoring was highly active in 2020 but declined in later years, with minor resurgence in 2023 and 2024. These early peaks likely reflect the foundational role of IoT-based data collection systems that support more advanced analytics downstream.

Prediction/Forecasting showed a clear upward trajectory, with activity growing steadily from 2020 (1 paper) to a peak in 2024 (6 papers). This progression indicates a shift toward model-based, anticipatory systems that leverage historical and real-time data for proactive decision-making.

Together, these trends point to a maturing research landscape—moving from sensor infrastructure and basic data reporting toward complex behavioral inference and predictive analytics in smart apiculture.

**Figure 9:**
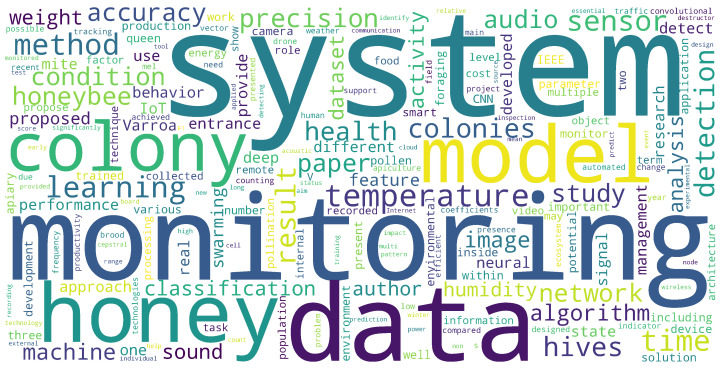
Word cloud of the most frequent terms appearing in abstracts of publications.

Figure 9 presents a word cloud generated from the abstracts of the reviewed studies, highlighting the most frequently occurring terms in smart beehive literature. Dominant keywords such as system, monitoring, data, honey, and colony reflect the field’s central focus on automated hive management and data-centric decision-making. The prominence of terms like monitoring and data reinforces the foundational role of sensor networks and continuous observation, while frequent mentions of honey and colony emphasize biological productivity and colony-level welfare as key research drivers.

#### 3.2.1. Sensors in Smart-Beehive Systems

**Figure 10:**
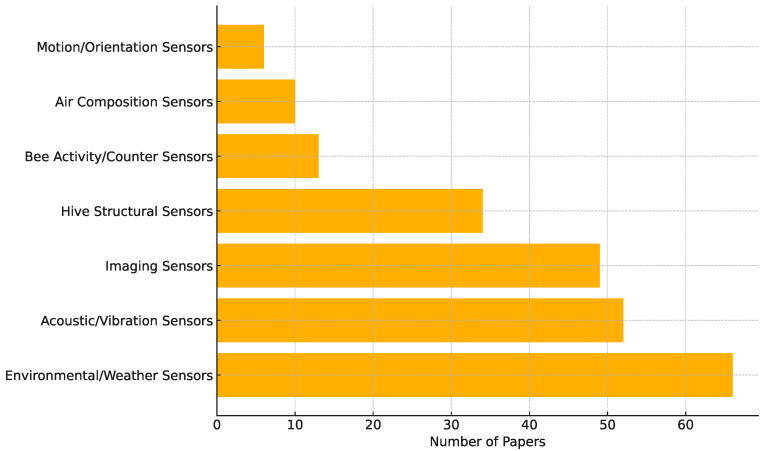
Prevalence of different sensor types in smart beehive studies.

The following subsections explore sensor modalities used in smart-beehive systems. A wide range of sensor types have been employed across the reviewed smart beehive. Figure 10 summarizes the distribution of sensor modalities by indicating how many of the 135 studies involved each sensor type. It is important to note that the presence of a sensor in a study does not necessarily imply that the sensor was physically deployed by the authors. In many cases, the researchers either collected their own data using such sensors or utilized publicly available datasets obtained from hives instrumented with the corresponding sensor types. Consequently, multiple sensors may be counted per study, and the totals in Figure 10 exceed the number of studies reviewed.

Several clear patterns emerge from Figure 10. Environmental sensors are the most commonly used, featured in 66 studies, which accounts for approximately 49% of the publications. This prevalence reflects the foundational importance of internal/external climate monitoring for assessing hive health and the relative ease of collecting such data [7,40,42,132].

Close behind are acoustic/vibration sensors, used in 52 studies, representing about 39% of the total. Their popularity underscores the value of hive sound patterns—acoustic signals provide insights into colony behavior, queen status, swarming tendencies, and stress indicators [43,48,53,84,90].

Imaging sensors appear in 49 studies, making up roughly 36% of the reviewed publications. The growth in computer vision methods, driven by increasingly accessible hardware and advances in image processing, has made visual analysis a leading choice for bee traffic monitoring and pathogen detection [10,81,82,110].

Hive weight/structural sensors are used in 34 studies, which corresponds to approximately 25%. This highlights the utility of weight measurements for tracking honey accumulation, food reserves, and colony strength with load cells or strain gauges [4,25,30,86].

More specialized sensor types are used less frequently. Bee activity counters appear in 13 studies, amounting to about 10%, and are often implemented through tags and cameras, infrared gates, or other entryway counters for quantifying foraging activity or ingress/egress patterns [47,49,54,109].

Air composition sensors are used in 10 studies, which represents roughly 7%. While potentially valuable for correlating gas levels with colony metabolism or health, their cost and limited interpretability may explain the relatively low adoption [76,88,96,133].

Finally, motion/orientation sensors are the least utilized, appearing in just 6 studies—approximately 4% of the total. Their use is typically limited to detecting external disturbances such as hive displacement due to wind, physical impact, or theft. As these events are relatively rare or peripheral to core hive monitoring objectives, such sensors are less commonly integrated into smart beehive systems [16,31].

**Figure 11:**
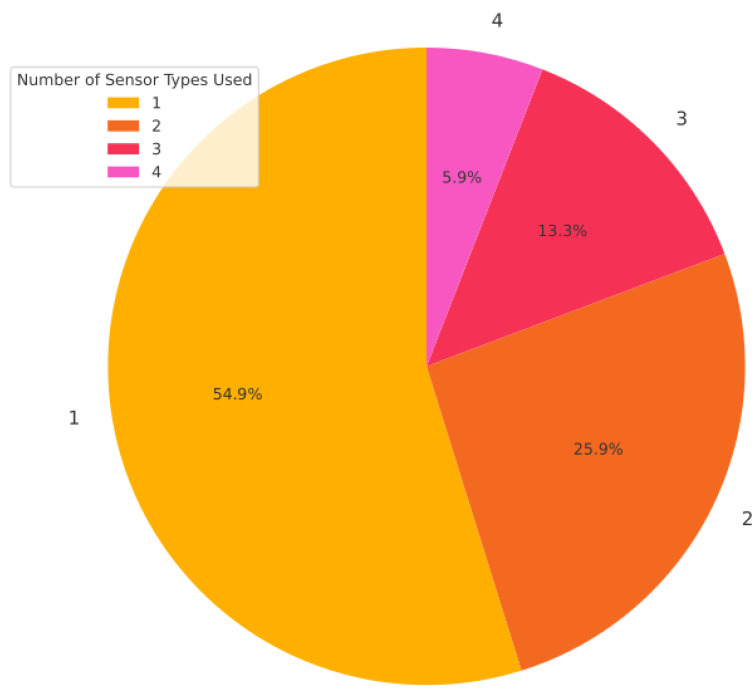
Distribution of smart hive studies by number of distinct sensor types used.

It is worth noting that the average smart hive study employs multiple sensor types to gain a more holistic view of the colony. In our dataset, systems used on average about 1.7 distinct sensor modalities each. As shown in Figure 11, about 55% of studies (74 out of 135) used exactly one sensor type—often these were single-modality systems such as purely acoustic or image monitoring setups. In contrast, approximately 45% of the studies employed a combination of two or more sensor types.

#### 3.2.2. Analytical Techniques and Algorithm Performance

After acquisition, sensor data must be processed and interpreted. This subsection summarizes the analytical methods and machine-learning algorithms used in smart beehive research, highlighting adoption trends and exemplary performance metrics.

A significant portion (26%) of studies integrated two sensor types, while 13% used three distinct modalities. Only a small fraction (6%) of projects incorporated four sensor types in a single system like [25,27,109]. This multi-sensor approach reflects the principle of sensor fusion: by combining complementary data sources, researchers can cross-validate findings and detect complex colony conditions that may not be evident through a single modality alone.

However, adding more sensors inevitably increases system complexity, cost, and power requirements—likely explaining why very few projects go beyond three or four sensing modalities.

**Figure 12:**
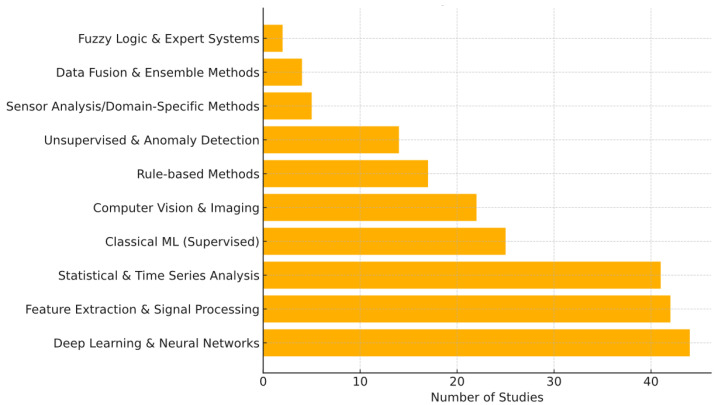
Frequency of various data analysis and machine learning method categories in the literature.

The intelligence in smart beehive systems comes from the data analysis algorithms that process sensor inputs into meaningful predictions, detections, or decisions. The reviewed studies span a broad spectrum of analytical approaches, from simple statistical thresholding to cutting-edge deep learning models. Figure 12 shows the distribution of major classes of AI/analysis methods used across the 135 studies (many studies employ more than one type of analysis, so counts are overlapping).

Deep learning and neural networks have become the most widely used analytical approach, with 44 studies accounting for about 33% of the corpus employing such techniques. This category includes the use of convolutional neural networks (CNNs) for image classification (e.g., identifying pests or classifying bee species/health from images) [58,87], as well as other neural network architectures for analyzing audio spectrograms or multivariate sensor time-series [98]. The prevalence of deep learning indicates that many researchers have started leveraging large datasets and powerful computing to improve detection accuracy in complex tasks like image-based mite detection or audio-based behavior classification. Indeed, deep learning often outperforms classical methods given sufficient data, which aligns with its adoption in about one-third of the studies.

Almost equally common are feature extraction and signal processing techniques, recorded in 42 studies, representing approximately 31% of the total. This category represents the foundational step in many analyses, for example, calculating the Fourier transform of audio to obtain frequency features [59] or performing image preprocessing and segmentation to isolate regions of interest (like bee or mite shapes in an image) [83]. Such techniques often precede other analyses and are pervasive as a component of the methodology, hence their high count.

Basic statistical and time-series analysis methods were employed in 41 studies, accounting for around 30%. These include approaches like correlation analysis [122], ARIMA models for time-series forecasting of hive parameters [121], or simple regression models [86]. Many papers rely on statistical analysis to interpret trends (for example, to see daily patterns in weight or temperature) or to set adaptive thresholds (like control charts for abnormal sensor readings). The substantial usage of statistical methods reflects that not all smart hive research relies on complex AI—sometimes straightforward statistical modeling suffices to derive insights from sensor data [80,126].

Turning to classical machine learning, supervised ML techniques appear in 25 studies, making up roughly 18% of the corpus. These methods include algorithms such as support vector machines, random forests, k-nearest neighbors, or naive Bayes classifiers. They have been applied, for instance, to classify sound patterns (using features like Mel-frequency cepstral coefficients from audio) [53], to distinguish normal vs. abnormal hive states [84], or to predict outcomes like swarm occurrence based on multivariate sensor inputs [13,14]. While classical ML is less dominant than deep learning in recent literature, it remains relevant, especially for moderate-sized datasets or where model interpretability is valued.

Applications of computer vision and image analysis techniques were noted in 22 studies, corresponding to approximately 16%. This category overlaps partially with deep learning because many vision tasks now use CNNs; however, it also covers conventional image processing (background subtraction, contour detection, etc.) and classical vision algorithms. The presence of computer vision methods reflects the significant subset of works dealing with camera data—counting bees at the entrance [10], detecting mite specks on bees [106], tracking bee motion in video [46], etc. Some studies combined traditional vision algorithms with newer ones (e.g., using feature detectors alongside CNNs to improve robustness [77]).

We also observed rule-based methods in 17 studies, which amounts to about 13% of the total. These are systems using predefined rules or logic, such as if-then rules triggered by sensor thresholds or expert system approaches. They tend to appear in early or simpler systems, for example, a system that sends an alert if weight drops by more than X in a day (indicating a swarm) or if temperature falls outside a band [3,34]. While conceptually straightforward and easy to implement, pure rule-based systems are less adaptive and may not handle complex patterns, which is likely why their relative usage has diminished over time in favor of learning-based methods.

A smaller portion of studies applied unsupervised learning or anomaly detection techniques, with 14 studies representing around 10%. These include clustering algorithms to group similar hive conditions, or outlier detection methods to flag unusual sensor patterns without pre-labeled examples. Such approaches are valuable when trying to detect novel or unexpected events (for instance, an unknown type of anomaly in hive sound or climate that was not specifically trained for) [15,124]. However, unsupervised methods require careful interpretation and have seen limited use, often complementing other analyses rather than being standalone solutions.

A smaller subset of studies employed other, less common analytical strategies. These include domain-specific methods, data fusion, ensemble methods, and fuzzy logic systems, collectively appearing in only a handful of cases. Such approaches often aim to integrate multiple data sources or model outputs, or to handle uncertainty through rule-based reasoning. While conceptually valuable—particularly for combining sensor modalities or managing ambiguity in biological systems—their limited presence likely reflects practical constraints such as small datasets, system complexity, or the dominance of more scalable data-driven techniques in the field [6,78,88,91,139].

**Figure 13:**
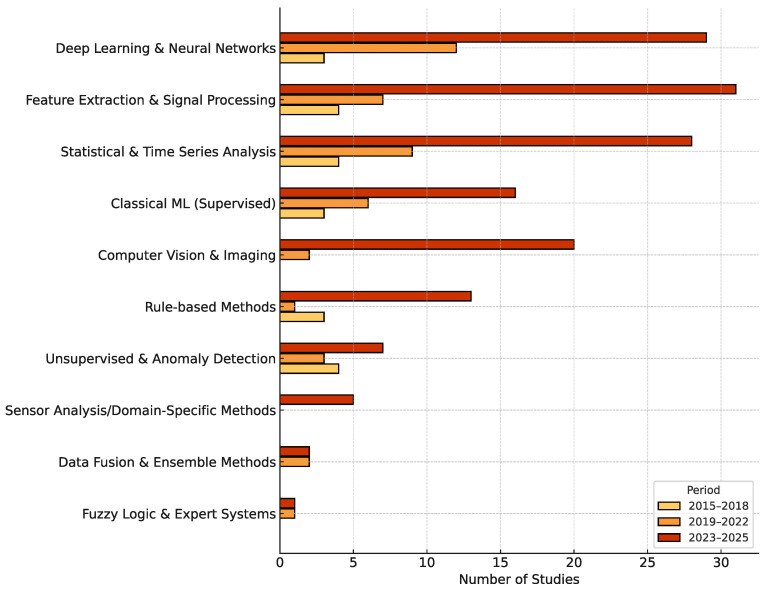
Temporal evolution of key data analysis methods used in smart beehive studies.

To understand the progression of analytical approaches in smart beehive research, Figure 13 displays the distribution of data analysis methods across three distinct periods: 2015–2018, 2019–2022, and 2023–2025. Table 4 complements this by summarizing the overall adoption rate of analytical techniques, including the period prior to 2015.

Before 2015, no studies employed analytical methods, reflecting the field’s early focus on hardware prototyping and sensor integration rather than data interpretation.

Between 2015 and 2018, 68.8% of studies utilized at least one analytical technique. This period was marked by methodological diversity rather than dominance, with various approaches each appearing in only a few studies. These early adopters explored a broad range of techniques, indicating a formative stage of experimentation without clear methodological convergence.

In the 2019–2022 period, the use of analytics increased to 81.2% of studies. Deep Learning and Neural Networks emerged as the most frequently used method, featuring in 12 studies—quadrupling its earlier usage. Statistical and Time Series Analysis and Feature Extraction and Signal Processing also gained traction, used in 9 and 7 studies, respectively. This indicates a shift toward more robust modeling strategies, particularly suited for capturing temporal patterns and supporting health diagnostics or forecasting applications.

By 2023–2025, analytical methods were nearly universal, applied in 95.3% of studies. Feature Extraction and Signal Processing led with 31 studies, followed closely by Deep Learning and Neural Networks (29) and Statistical and Time Series Analysis (28). Notably, Computer Vision and Imaging methods rose sharply to 20 studies, reflecting increased emphasis on visual behavior tracking and swarm detection. Meanwhile, Classical ML (Supervised) maintained stable adoption (16 studies), suggesting it is being complemented or gradually supplanted by more advanced architectures. This period marks the transition toward integrated, multimodal, and predictive systems in smart apiculture.

Together, these trends highlight the maturation of smart beehive research—from early-stage experimentation to a data-driven discipline where neural networks, signal processing, and vision-based analytics play a central role in colony monitoring and decision-making.

#### 3.2.3. Comparative Assessment of Sensors and Algorithms

We synthesized quantitative comparisons of commonly used sensors and machine learning approaches. Table 5 and Table 6 summarize representative sensor characteristics (accuracy and approximate cost) and published performance metrics for exemplary algorithms.

### 3.3. Meta-Analysis of Publications

An interesting question is how the choice of sensor modalities correlates with the research goal of a study. Different application domains might favor certain sensors. To explore this, we present several heatmaps and discuss the patterns they reveal.

#### 3.3.1. Sensor Usage by Research Objectives

**Figure 14:**
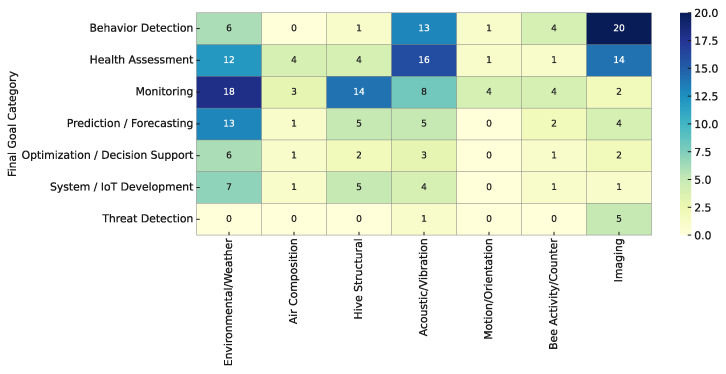
Heatmap showing the usage frequency of each sensor modality within each main goal category of studies.

Figure 14 shows the co-occurrence of sensor types with each main goal category, highlighting patterns of association between sensing modalities and research objectives.

Several clear patterns can be observed in Figure 14: Behavior Detection studies heavily rely on imaging and acoustic sensors. This category includes the highest concentration of works utilizing cameras or visual tracking, along with a strong presence of studies using acoustic sensing. This aligns with expectations—analyzing bee behaviors such as foraging or in-hive activity often depends on direct observation through visual or auditory cues.

Health Assessment studies make substantial use of acoustic and imaging sensors, as many health diagnoses involve detecting anomalies in either sound—such as changes in buzzing from sick colonies—or visual patterns, like images of bees or brood used to identify mites or disease symptoms. These studies also frequently incorporate environmental sensors, since temperature shifts may signal brood issues or colony decline, and certain diseases can manifest through subtle changes in microclimate. A small subset of health-focused research has explored the use of air composition sensors to detect chemical markers of illness or elevated CO_2_ resulting from poor ventilation in weakened hives. On the other hand, structural sensors such as those measuring hive weight appear less commonly in this context, as weight is generally more associated with food reserves than with disease—although significant weight loss can still indicate potential problems. Overall, health-monitoring systems tend to be multimodal, designed to detect a broad spectrum of physiological and behavioral symptoms.

Monitoring studies, as expected, prioritize the use of environmental and hive structural sensors. These general-purpose platforms often focus on recording vital hive parameters such as internal and external temperature and humidity, air pressure, wind speed, and hive weight. Their goal is usually to provide a comprehensive overview of hive status by capturing key physical indicators. Some studies also incorporate acoustic sensors to detect sound-related anomalies, such as sudden silence following colony collapse or excessive noise indicating disturbance.

Prediction and Forecasting studies frequently rely on environmental data, along with a notable presence of structural and acoustic inputs. For example, models predicting honey yield often use weather conditions and historical weight trends, while forecasts of swarming behavior might incorporate temperature and sound cues. The strong emphasis on environmental parameters is logical, given that many colony events—like nectar flow or swarm triggers—are closely tied to seasonal and weather-related patterns.

The remaining categories—Optimization and Decision Support, System and IoT Development, and Threat Detection—exhibit more specific sensor usage patterns, reflecting their narrower focus or earlier stage of maturity. Optimization-focused studies, although limited in number, typically employ environmental sensors to support yield improvement or management recommendations. System development papers consistently incorporate environmental, and frequently also structural and acoustic, sensors—suggesting that a standard sensing suite (e.g., temperature, humidity, weight, sound) is considered essential when building general-purpose platforms. In contrast, imaging sensors are less common in this group due to power and complexity constraints. Meanwhile, threat detection studies rely almost exclusively on imaging, sometimes complemented by acoustic sensing, to visually identify external aggressors such as hornets. The absence of environmental and weight sensors in these works highlights the highly targeted nature of this application domain.

These correlations underscore that the selection of sensors in a smart beehive project is closely aligned with its objectives. If the goal is to monitor or predict general hive status, environmental and weight sensors are the go-to choices for tracking broad trends. For researchers and engineers, this insight can guide the design of future systems: depending on the primary application, one can prioritize certain sensor modalities to maximize the likelihood of success.

#### 3.3.2. Sensor Co-Occurrence Patterns

**Figure 15:**
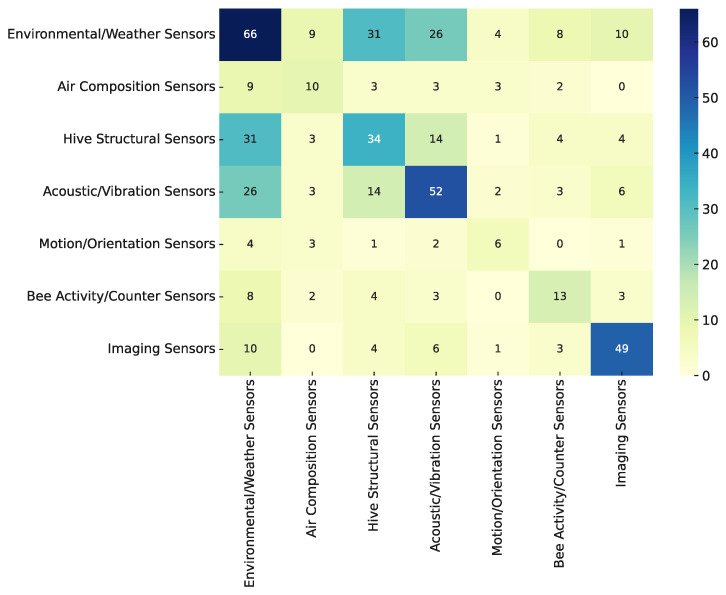
Sensor modality co-occurrence matrix across smart beehive studies.

The sensor co-occurrence heatmap shown in Figure 15 provides a quantitative overview of how different sensor modalities are jointly used in smart beehive systems.

The most prominent co-occurrence is observed between environmental and hive structural sensors. This pairing reflects a foundational design in beekeeping technology, where ambient conditions such as temperature and humidity are monitored in parallel with hive weight to assess colony growth, honey production, or seasonal changes. In many cases, the weight sensor serves as a proxy for biomass or food reserves, while temperature provides thermal context—although the two data streams are often analyzed independently.

Another commonly observed combination involves environmental sensors and acoustic/vibration sensors. These systems typically use microphones or accelerometers to monitor hive activity, agitation, or swarming behavior, while simultaneously recording internal/external temperature. However, the data are frequently interpreted in isolation: acoustic features are used for classification or anomaly detection, whereas temperature readings are either passively logged or used to validate overall hive conditions.

A moderately common pattern includes the co-occurrence of hive structural and acoustic/vibration sensors, a setup that theoretically enables the study of behavioral states in relation to hive mass or movement. Similarly, the combination of environmental sensors with imaging systems is often found in platforms that monitor entrance traffic or thermal vision, though true fusion of visual and thermal features remains rare.

Other co-occurrences—such as those involving bee activity counters, motion/orientation sensors, or air composition sensors—are less frequently encountered and tend to serve more specialized roles. For instance, gas sensors like CO_2_ or O_2_ are typically used to investigate hive respiration or ventilation, while accelerometers may be included for structural monitoring or theft detection. These modalities are rarely integrated with others in a unified analytical framework, although there are notable exceptions. Newton et al. [96], for example, combine CO_2_, temperature, vibration, and weight data to infer colony behavior during overwintering. Similarly, Robustillo et al. [111] apply vector autoregressive models to jointly analyze temperature, humidity, weight, and meteorological variables for predicting internal hive conditions. Henry et al. [1] also examine variability in acoustic and environmental data to assess colony stress under electromagnetic exposure, suggesting potential for integrated interpretations. In another example, the b+WSN platform [14] incorporates gas, temperature, and weight sensors into a rule-based decision model that triggers alerts at the hive level.

A key insight drawn from the literature is that while sensor integration at the hardware level is common, true multimodal analysis or data fusion remains largely absent. Even in more complex systems that include multiple sensor types, data from each source are typically processed in isolation. As such, the co-occurrence heatmap primarily reflects design choices and hardware configurations rather than analytical integration.

#### 3.3.3. Sensor–Model Co-Occurrence Patterns

**Figure 16:**
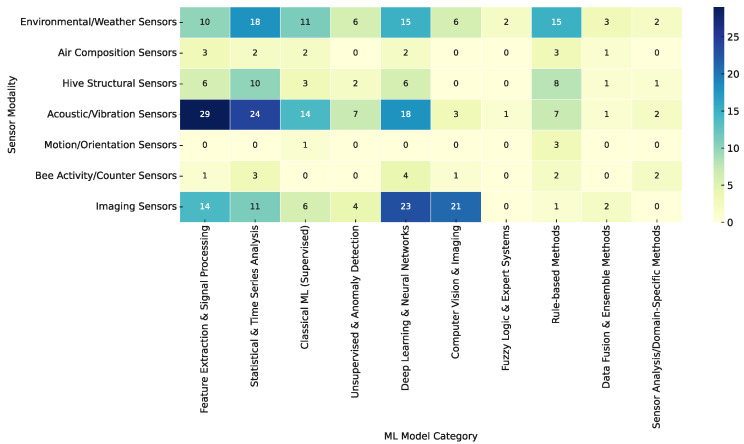
Heatmap showing the co-occurrence between sensor modalities and machine learning (ML) model categories across surveyed smart-beehive systems.

The sensor–model co-occurrence heatmap, shown in Figure 16, reveals several dominant patterns in the design of smart beehive systems, each reflecting how particular sensor modalities are suited to specific types of analysis as dictated by the intended application goals.

One of the strongest associations is between Acoustic/Vibration Sensors and a diverse range of analytical methods, including Feature Extraction and Signal Processing, Statistical and Time Series Analysis, Classical ML (Supervised), and Deep Learning and Neural Networks. This pattern reflects a substantial body of research focused on monitoring colony behavior, detecting stress responses, and identifying anomalies through audio-based cues. These systems typically rely on time–frequency representations of hive sound, such as spectrograms, which are then processed using machine learning or deep learning models. The focus in these works is often on non-invasive, real-time diagnostics aimed at swarm prediction or general hive health assessment.

Environmental/Weather Sensors are frequently paired with Statistical and Time Series Analysis. These studies generally seek to model colony microclimate, identify seasonal or daily patterns, and detect deviations from thermal norms that may indicate brood disturbance or weakening colonies. Given the scalar and temporal nature of the data, statistical methods such as trend analysis or control charts are both practical and interpretable, especially for low-cost, field-deployable systems.

Imaging Sensors show strong co-occurrence with Deep Learning and Neural Networks and Computer Vision and Imaging. These combinations are common in studies targeting automation of visual tasks such as bee counting, motion tracking, or disease detection based on visual symptoms. Convolutional neural networks (CNNs) dominate this space due to their ability to learn hierarchical visual features. Such systems are typically high-precision and are designed for monitoring hive entrance activity, external threats, or internal brood conditions.

Hive Structural Sensors, such as those used to measure weight, are most often linked with Statistical and Time Series Analysis. These systems often aim to infer honey production rates, colony strength, or feeding patterns by analyzing trends in weight data. Because weight is a cumulative and slowly varying signal, it naturally lends itself to forecasting models such as regression or ARIMA.

Other sensors—including Air Composition Sensors, Motion/Orientation Sensors, and Bee Activity/Counter Sensors—appear much less frequently and are mostly found in exploratory or proof-of-concept studies. Their limited analytical pairing reflects either the novelty of their application or the challenges of integrating their outputs into broader data pipelines. While some of these modalities show promise, their adoption remains sparse.

Overall, the co-occurrence analysis reveals that sensor selection and model choice are closely aligned with the nature of the data and the functional goals of the system. Acoustic and imaging data, being high-dimensional and temporally dynamic, are typically matched with learning-based models capable of complex pattern recognition. In contrast, environmental and structural data, which are scalar and trend-oriented, are more often analyzed using statistical or threshold-based techniques.

#### 3.3.4. Sensor–Communication Co-Occurrence Patterns

**Figure 17:**
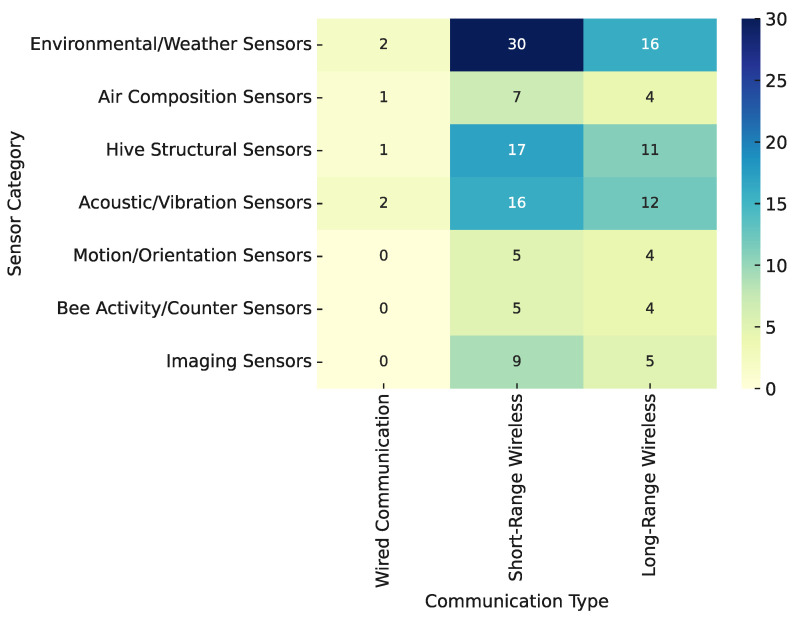
Co-occurrence between sensor categories and communication types across reviewed smart beehive systems.

Understanding how different sensor modalities are implemented and transmitted is crucial for designing efficient and scalable smart beehive systems. Various sensor types require distinct communication strategies depending on factors such as data rate, energy consumption, and deployment context. Figure 17 provides an overview of the co-occurrence patterns between sensor categories and communication technologies used in the reviewed literature. This visual summary helps illustrate which sensor types are commonly paired with wired, short-range, or long-range wireless communication, highlighting both standard practices and emerging trends in smart hive design.

Environmental/Weather Sensors, Hive Structural Sensors, and Acoustic/Vibration Sensors are most frequently paired with Short-Range Wireless and Long-Range Wireless communication technologies. This common pairing reflects their central role in smart beehive systems, where real-time or continuous data—such as temperature, humidity, hive weight, or sound—must be transmitted efficiently from remote or outdoor environments. The low data rate and power requirements of these sensors make them particularly suitable for low-energy wireless protocols. As a result, they are widely adopted in both experimental setups and field-deployable platforms, offering a balance between communication efficiency, scalability, and monitoring reliability.

Imaging Sensors are more selectively paired, predominantly with Short-Range Wireless communication. This likely reflects their high data bandwidth requirements, which are better handled with nearby base stations or local edge processing units. These sensors often appear in vision-based systems focused on tasks such as bee counting, foraging activity monitoring, or intrusion detection at the hive entrance.

Across all sensor types, Wired Communication is used infrequently, primarily in early-stage prototypes or laboratory settings where simplicity and data stability are prioritized over deployment flexibility. Most modern implementations prefer wireless connectivity, reinforcing the field’s emphasis on modularity, scalability, and field-readiness.

In summary, the communication strategy in smart beehive systems is closely aligned with sensor function, data rate requirements, and deployment context. Foundational sensors like environmental and structural ones appear across a wide range of systems and communication protocols, while high-bandwidth or specialized sensors show more constrained and deliberate communication pairings.

### 3.4. Practical and Technical Limitations

A cross-study analysis of recent literature reveals a variety of practical and technical limitations that hinder the deployment, reliability, and scalability of smart beehive monitoring systems. These challenges arise across multiple layers—from data collection and algorithm design to hardware constraints and environmental conditions.

One major limitation is the availability and quality of data. Many studies report small dataset sizes and a lack of environmental diversity, making models vulnerable to overfitting and poor generalization. Edwards-Murphy et al. [14] and Braga et al. [79] highlight issues such as the absence of representative samples for different hive states and the geographic confinement of data collection. Žgank [52] and Campell et al. [124] emphasize that insufficient variability in training data can severely impact model performance—for instance, by causing convergence to trivial identity matrices in swarm detection methods based on matrix factorization. In addition, Gil-Lebrero et al. [8] point out that the inherent biological variability in beehive activity introduces further inconsistency into datasets.

On the algorithmic side, many models rely on computationally intensive methods such as deep learning or spectral decomposition, which often exceed the capabilities of resource-constrained edge devices used in field settings. Kulyukin et al. [44] note that deep models demand significant processing power, limiting their real-time deployment potential. Campell et al. [124] raise additional concerns about convergence behavior in spectral methods, while Kulyukin et al. [121] describe how sensor faults and environmental disruptions can create discontinuities in time-series data, degrading the reliability of forecasting models. Cecchi et al. [27] also report performance limitations in vision-based systems due to segmentation errors.

Hardware, energy, and communication constraints present further obstacles. Solar-powered hives often fail to harvest sufficient energy for continuous monitoring, as observed by Edwards-Murphy et al. [22]. Scalability is another concern—Kviesis et al. [37] report that their system could securely support only ten IoT nodes. High costs and lack of flexibility in commercial platforms limit their adaptability in field conditions, as pointed out by Hamza et al. [39]. Other authors [28,135] note that general-purpose computing platforms are often unsuitable due to their energy inefficiency and lack of durability. Multiple studies [13,22] independently report the inadequacy of solar energy harvesting, indicating this is a widespread challenge.

Environmental sensitivity adds another layer of complexity. Sensor placement within the hive can significantly affect measurement accuracy—Catania and Vallone [29] demonstrate that temperature readings vary depending on probe location. Lighting conditions, occlusion, and hive structure all affect the reliability of visual data [2,27], illustrating how fragile sensor performance can be in uncontrolled environments.

Lastly, the maturity of many systems remains limited. Numerous solutions are still in early-stage or prototype phases. Kulyukin and Mukherjee [44] provide only preliminary evaluations of their models, and Szczurek et al. [76] explicitly call for further validation of gas-based detection techniques. The absence of long-term, multi-seasonal field testing makes it difficult to assess whether these systems can maintain reliability under natural variability and operational stress.

Taken together, these limitations reflect the growing pains of a research field still transitioning from proof-of-concept studies to practical, field-ready technologies. Addressing them will require robust datasets, computational efficiency, resilient hardware, and sustained validation efforts.

## 4. Publicly-Available Datasets for Smart-Beehive Research

Effective machine learning models for monitoring honey bee colonies rely on access to structured, labeled datasets that reflect the complexity of hive dynamics across multiple sensing modalities. Accordingly, to address our final research question (RQ5) on data resources, we survey the publicly available datasets that have been used to support smart beehive studies. Over the past several years, a number of high-quality public datasets have emerged, capturing audio signals, visual observations, and environmental telemetry relevant to colony health and behavior. These resources support tasks such as swarm prediction, parasite detection, behavior classification, and vitality forecasting. A summary of the most prominent datasets, categorized by modality and typical machine learning application, is provided in Table 7.

### 4.1. Acoustic Datasets

Acoustic monitoring offers a non-invasive method for assessing beehive conditions, providing valuable insights into colony behavior and health.

The “To bee or not to bee: An annotated dataset for beehive sound recognition” dataset, created by Inês Nolasco and Emmanouil Benetos from Queen Mary University of London [141], focuses on the automatic recognition of beehive sounds. This dataset is composed of 78 recordings, totaling approximately 12 h of audio, sourced from the Open Source Beehive (OSBH) project and the NU-Hive project. The audio segments are primarily labeled into two classes: “Bee” (pure beehive sounds) and “noBee” (periods where external sounds are perceived, superimposed on bee sounds). Annotation was performed by volunteers using Sonic Visualiser, leveraging both auditory perception and visual analysis of log-mel frequency spectrums. This dataset is explicitly designed for investigating machine learning approaches to beehive sound recognition and evaluating developed methods. A related dataset, “Audio-Based identification of Beehive states: The dataset” created by Ines Nolasco, Alessandro Terenzi, Stefania Cecchi, Simone Orcioni, Helen L. Bear, and Emmanouil Benetos [142], also contains audio files and a state_labels.csv for the audio-based identification of beehive states. Another publicly available beehive audio dataset contains 10,000 audio files, each 8.203125 s long, sampled at 8000 Hz in WAV format, identified by date, time, and hive ID [152]. Another relevant source is the “Smart bee colony monitor: Clips of beehive sounds” dataset published on Kaggle by Anna Jyang [143]. It includes multiple recordings of beehive audio categorized by labels such as “healthy”, “distressed”, and “empty”. The dataset serves as a foundation for machine learning applications focused on recognizing bee colony states through sound analysis, and complements existing audio datasets in offering class-labeled audio in various beehive conditions.

The “Dataset for honey bee audio detection,” by Pawel Biernacki from the University of Science and Technology Wroclaw [18], provides 10,000 one-second recordings of bees and 1700 one-second recordings of drones. All recordings are in WAV format without compression, sampled at 44.1 kHz. The specific labeling of “bees” and “drones” makes this dataset directly applicable for developing and evaluating ML models for audio detection and classification of different honey bee types.

The “Queenless honeybee acoustic patterns” dataset, contributed by Antonio Robles-Guerrero [144], contains acoustic patterns from five Carniola honeybee colonies in Zacatecas, Mexico. The dataset includes recordings from healthy queenright colonies (with huge and moderate populations) and queenless colonies (with low populations), established by removing queens from two colonies. Each sample is 30 s long, recorded at a sampling frequency of 4 kHz with 12-bit resolution. The explicit hypothesis is that the queenless state can be identified by comparing acoustic patterns with healthy colonies using machine learning techniques and feature extraction methodologies.

### 4.2. Visual Datasets

Visual data provides direct observational insights into bee activity, health, and interactions within the hive environment.

The “Labeled dataset for bee detection and direction estimation on beehive landing boards,” contributed by Tomyslav Sledevic [145], includes several visual datasets:

- A detection dataset with 7200 frames (1920 × 1080 resolution) for bee detection/ segmentation.
- A segmentation dataset with 2300 cropped bee images labeled with a triangle shape for direction vector estimation.
- A pose directory containing 400 frames from eight beehive entrances, where annotations include two points (head and stinger, or front and back if partially visible) for bee direction estimation.
- A ramp detection dataset with 156 images, annotated with bounding box coordinates and four keypoints.
- Tracking and behavior datasets consist of annotated MP4 files with bee tracks during foraging, defense, fanning, and washboarding activities within the entrance zone.

All annotations are in YOLO format, supporting the development of ML models for object detection, segmentation, pose estimation, tracking, and classification of specific bee behaviors.

The “Dataset for varroa mite detection on sticky boards,” created by Jose Divasón et al. [146], provides 64 high-resolution images (8064 × 6048) of sticky boards with *Varroa mites*, along with their labels. The dataset also includes a version of these images after deblurGAN techniques have been applied. This dataset is intended for use with deep learning techniques to analyze Varroa mite colony infestation levels and includes predefined training and validation splits, as well as developed deep learning models.

The “VarroaDataset” developed by Schurischuster Stefan and Martin Kampel [17], offers high-resolution images (160 × 280 px) of honeybees, specifically focusing on the presence of the *Varroa destructor* parasite. The dataset contains 13,509 samples, with approximately 3947 manually annotated as infected (class 1) and 9562 as healthy (class 0). It includes predefined dataset splits for training, testing, and validation. Bounding box coordinates are provided for the annotations. This dataset is explicitly designed for detecting parasites on honeybees using machine learning, particularly for image classification and object detection tasks.

The “VnPollenBee Dataset” is specifically built for detecting pollen-bearing bees from videos captured at hive entrances [147]. It comprises over 2000 images, manually annotated, containing 1758 pollen-bearing bees and 59,068 non-pollen-bearing bees. The images were extracted from 1920 × 1080 resolution videos recorded at 60 frames per second under varying natural light conditions. Annotations were initially manual using Labelme Annotation tools and refined with an object detection model. The dataset is pre-divided into training, validation, and test sets (70:20:10 ratio) to facilitate comparative studies of deep learning models for pollen bee detection. The “The BeeImage dataset: Annotated honey bee images” dataset on Kaggle, created by Jenny Yang [148], provides a collection of annotated bee images aimed at object detection tasks. The dataset consists of over 1000 labeled images with bounding boxes around honey bees, intended to facilitate training and evaluation of deep learning models for detection and classification tasks. It is particularly useful for preliminary experimentation in object detection pipelines.

### 4.3. Environmental and Multimodal Datasets

These datasets combine various sensor measurements to provide a comprehensive understanding of beehive dynamics and their external influences.

A bee colony monitoring system, detailed in the study “Bee colony remote monitoring based on IoT using ESP-NOW protocol” collected real-time environmental data [37]. The study makes available the “Measurements for the experimental period” dataset as supplemental information. This dataset includes battery discharge rates, temperature measurements (inside and outside the hive), and weight measurements of bee colonies. This data, collected from five colonies in Latvia from June to August 2022, was used to evaluate the efficiency of the IoT system and to analyze colony weight dynamics for active foraging periods, as well as in-hive temperature for colony state assessment. Such data is fundamental for developing ML models for predictive monitoring of colony health, activity levels, and resource availability.

The “Research project on field data collection for honey bee colony model evaluation—datasets” (also known as the MUSTB field data collection), created by Dupont Yoko L. et al. [149], provides a comprehensive set of data for evaluating honey bee colony models [149]. It includes various data modalities:

- Environmental/Physiological data, such as hive weight obtained from automatic logging by a hive scale, and adult bee strength from weight assessment of combs.
- Visual data, including data on brood development and food provision from image analysis of combs, and forager activity from automatic video recordings and image analysis by a bee counter.
- Observational and management logs, detailing colony management actions (e.g., input/output of materials, queen loss, swarming, clinical signs) and observations of honey bee waggle dances (orientation and direction).
- Chemical and biological analysis results, including laboratory analyses of pollen, pesticide residues, and parasites/pathogens.
- Geographical information for sites and polygons, including UTM coordinates.

The data is reported according to a specific data model and stored in a relational database, providing a rich, multimodal resource for developing and evaluating diverse machine learning models related to bee colony health, behavior, and environmental interactions.

The “Winter carbon dioxide measurements in UK honeybee hives 2022/2023” dataset, contributed by Michael Newton [150], specifically reports carbon dioxide measurements in wintering beehives in the UK. This data is also compared with hive scale and vibration sensor measurements. While not explicitly detailing ML model use within the source, CO_2_ levels, hive mass, and vibration patterns are crucial environmental indicators that can be used to train and evaluate ML models for assessing colony vitality, wintering success, and overall health without manual inspection. This data supports “Giving Beekeeping Guidance by computational-assisted decision making,” implying its relevance for ML-driven decision support systems.

Although not directly a beehive-specific dataset, NASA POWER (Prediction Of Worldwide Energy Resources) provides publicly accessible solar and meteorological datasets [151]. The “Agroclimatology Archive” specifically targets agricultural needs and provides parameters formatted for input to crop models. While it does not contain bee-specific labels, this external environmental data, such as temperature, solar radiation, and other meteorological parameters, is highly relevant for smart-beehive research. It can be integrated into ML models to contextualize bee behavior, foraging patterns, and colony health responses to broader environmental conditions.

### 4.4. Summary

In summary, a growing number of publicly available datasets are instrumental in advancing smart-beehive research. These datasets offer diverse data modalities, including acoustic signals for sound recognition and queen state detection, visual imagery for bee and parasite detection, and a range of environmental and physiological measurements for comprehensive colony monitoring. The detailed labeling and structured organization of these datasets directly support the development, training, and evaluation of various machine learning models for tasks such as classification, object detection, pose estimation, tracking, and behavioral analysis, ultimately contributing to more effective precision apiculture.

## 5. Discussion and Future Work

The findings from this review illustrate the remarkable progress made in integrating sensing, communication, and AI technologies into beekeeping. However, a closer analysis reveals a number of systematic limitations that, if addressed, could lead to significantly more robust, scalable, and intelligent smart hive systems.

### 5.1. Sensor Modalities and Deployment Gaps

Environmental sensors—particularly those for temperature, humidity, and hive weight—remain the most commonly deployed modalities due to their affordability and ease of integration [2,4,8]. Acoustic sensors rank second and are widely used for non-invasive detection of hive events such as queen loss or swarming [1,144,152]. These audio-based methods have proven particularly effective for identifying critical changes in colony behavior.

However, other sensor types remain underutilized despite their potential value. Gas sensors such as CO_2_ and NO_2_ sensors can offer insight into hive respiration and ventilation patterns [153], while infrared imaging and tag-based bee counters can provide information on thermal dynamics and foraging rates [141,148]. Despite their promise, few reviewed systems integrated these additional modalities, indicating a narrow focus in current experimental designs.

Moreover, most systems use only a single sensor modality, which limits resilience in noisy or uncertain environments. Multimodal sensor fusion—where acoustic, environmental, and even image-based signals are combined—remains rare in practical deployments despite its proven benefit in robustness and accuracy [3,145]. Furthermore, calibration protocols, sensor placement standards, and long-term durability studies are seldom reported, which impairs reproducibility.

### 5.2. Novel Opportunity: Signal-Layer Metrics as Passive Sensors

A promising research direction for smart beehive systems is the use of wireless communication signal metrics—such as Received Signal Strength Indicator (RSSI) and Signal-to-Noise Ratio (SNR)—as low-cost, passive sensing modalities. These metrics, inherent to radio communication technologies like LoRaWAN, can exhibit environmental sensitivity and offer insights without requiring dedicated physical sensors.

Recent studies have demonstrated that fluctuations in signal strength can be correlated with changes in environmental parameters, such as soil moisture [154], occupancy and shadowing effects [155], or spatial positioning [156]. In these applications, RSSI and SNR patterns are interpreted using classical and machine learning techniques to infer states that would traditionally require more expensive and power-consuming sensors.

Applied to apiculture, similar principles could be exploited. For instance, changes in hive weight, bee clustering behavior, or humidity buildup may impact the wireless signal propagation between nodes. This opens the door to designing low-power, low-cost hives that leverage communication signals not just for data transmission, but also as sensing elements. Given that many smart beehive platforms already include long-range communication modules, signal-layer analysis could yield significant savings in hardware complexity and power consumption.

To our knowledge, no reviewed papers apply such differential signal-based sensing in beekeeping. However, the approach is promising due to its low power requirements, passive nature, and ability to integrate with existing LoRaWAN deployments. This form of “virtual sensing” could be especially valuable in constrained deployments and represents a novel research opportunity with wide applicability. We recommend future studies examine RSSI/SNR sensitivity to key beehive conditions, explore training ML models on such features for anomaly detection, and benchmark their accuracy against conventional sensors.

### 5.3. Data Processing and Machine Learning Approaches

The review also shows a clear evolution from rule-based alert systems to ML-powered classification and prediction. Traditional ML models like decision trees, support vector machines, and random forests are widely adopted for swarm prediction and audio classification tasks [6,15]. Deep learning models—including CNNs and LSTMs—are increasingly being used for vision and time-series inference [14,16,148].

Nonetheless, several methodological shortcomings were identified. First, the majority of models are trained on small or private datasets, reducing reproducibility and generalizability [142,147]. Second, comparative model evaluation using standard metrics is rare, making it difficult to benchmark performance. Finally, and most notably, very few systems implement TinyML—machine learning designed to run on microcontrollers—for real-time inference on edge devices.

This lack of on-device inference is a missed opportunity, particularly for remote apiaries with limited connectivity. TinyML models can process acoustic signals, environmental data, and even signal-layer features like changes in signal strength locally, enabling real-time decisions without requiring constant uplink.

### 5.4. Deployment and Reproducibility Challenges

Despite their technical promise, many reviewed systems were only validated in laboratory settings or over short time intervals [2,4]. Very few reported long-term, in-situ deployments that accounted for seasonal or geographic variation [149,157]. As a result, many systems remain proof-of-concept rather than field-ready solutions. Concrete integration challenges reported in the literature include (i) load-cell weight scales requiring rigid, level platforms and weatherproof housings to maintain ±0.1 kg accuracy; humidity and temperature fluctuations can drift calibration over time and necessitate periodic recalibration; (ii) microcontroller and sensor enclosures suffering condensation and propolis buildup in harsh beehive environments, leading to sensor failure; (iii) limited power budgets for wireless modules—long-range radios such as LoRa require careful duty-cycling or solar power to avoid battery depletion; and (iv) network latency and packet loss when multiple hives share a gateway, complicating real-time anomaly detection. These examples highlight that system engineering constraints, not just algorithmic performance, often limit the robustness and scalability of smart hive prototypes.

Reproducibility is another critical issue. Fewer than 10% of the reviewed papers provide access to source code or raw datasets [17,18]. Even when data is shared, it is often poorly labeled or lacks critical metadata, preventing meaningful reuse or comparison. The absence of benchmark datasets impedes progress and creates artificial barriers to entry for new researchers, as well as broader scientific insights into honey bee colony dynamics.

### 5.5. Future Research Directions

To advance the field, we recommend the following concrete actions:

- Design and deploy multimodal sensing platforms that combine multiple sensor types, and communication-layer signals (RSSI, SNR) for holistic hive monitoring.
- Explore fluctuations in signal strength using internal vs. external LoRaWAN nodes as a novel passive anomaly detection method.
- Develop lightweight, interpretable TinyML models capable of real-time inference on embedded microcontrollers using features like sound patterns, temperature, and RSSI fluctuations.
- Standardize data annotation, sharing, and benchmarking protocols through the creation of open-access, multi-season, multi-location datasets.
- Investigate privacy-preserving distributed learning techniques such as federated learning to enable collaborative model training across apiaries.
- Foster stronger collaboration with domain experts (experienced beekeepers and entomologists) to ensure smart beehive solutions address practical beekeeping needs and scientific knowledge gaps. This includes emphasizing user-friendly designs, cost-effectiveness, and validation of technologies in real-world apiary conditions.

By addressing these gaps, the community can transition from fragmented, lab-scale studies to robust, reproducible, and scalable smart hive systems capable of supporting both commercial and ecological beekeeping practices, as well as advancing scientific understanding of honey bee colonies.

## 6. Conclusions

This review systematically analyzed 135 peer-reviewed papers on smart beehive systems, identifying major technological trends, challenges, and research opportunities in the domain of precision apiculture. Environmental and acoustic sensors were found to be the most frequently used, while visual and gas sensing remain underexplored. Communication architectures favor short-range wireless protocols, though long-range low-power options like LoRa and NB-IoT are increasingly adopted. Methodologically, a transition is underway from rule-based systems to machine learning, though deep learning remains limited by data availability and deployment complexity.

The study reveals key gaps in sensor fusion, data transparency, and longitudinal validation. Addressing these will be crucial for the development of robust, scalable, and reproducible smart hive platforms. Future systems must emphasize multimodal sensing, edge intelligence, and open science principles. By consolidating existing work and outlining clear directions for research, this paper contributes a foundational synthesis for scientists, engineers, and beekeepers seeking to harness technology for sustainable apiculture.

## Figures and Tables

**Table 1:** Eligibility criteria for systematic review of smart beehive technologies.

Criteria	Description
Type of Data	Studies must report on environmental, acoustic, visual, or multisensory data collected from within or around beehives, supporting sensor-based monitoring or data-driven analysis.
Algorithms or Techniques	While not a mandatory component, the adoption of data-driven approaches is widely considered advantageous for deriving structured insights from sensor observations and facilitating evidence-based interpretations in smart beekeeping research.
Comparator	RQ1: Types of sensor modalities used. RQ2: Application domains. RQ3: Categories of ML and analytical methods used and trends in their adoption over time. RQ4: Reported technical and practical limitations, including system cost, data quality, power consumption, and deployment challenges. RQ5: Usage of publicly available datasets, categorized by data modality, labeling approach, and their role in model training or evaluation.
Outcome	Detailed characterization of smart beehive systems, including sensor setups, communication methods, ML/AI techniques, goals and reported limitations.
Timing	Articles published from January 1990 to April 2025.
Environmental or Geographical Context	No restrictions; studies from any geographic region are considered.
Publication Type	Peer-reviewed journal articles and conference papers published in English.

**Table 2:** Search strategy and number of retrieved records per database.

Database	Search Query
Web of Science	ALL = (( (precision OR smart OR intelligent OR automated) AND (beekeeping OR beehive OR apiculture OR apiary) ) OR “precision beekeeping” OR “smart beehive”) AND DT==(“ARTICLE” OR “PROCEEDINGS PAPER”) AND DOP=1990-01-01/2025-04-07
IEEE Xplore	(“All Metadata”:“precision beekeeping” OR “All Metadata”:“smart beehive” OR ( (“All Metadata”:“precision” OR “smart” OR “intelligent” OR “automated”) AND (“All Metadata”:“beekeeping” OR “beehive” OR “apiculture” OR “apiary”) ) ) AND (“ContentType”:“Journals” OR “ContentType”:“Conferences”)
Scopus	TITLE-ABS-KEY( ( ( precision OR smart OR intelligent OR automated ) AND ( beekeeping OR beehive OR apiculture OR apiary ) ) OR “precision beekeeping” OR “smart beehive” ) AND PUBYEAR > 1990 AND ( LIMIT-TO ( DOCTYPE,“ar” ) OR LIMIT-TO ( DOCTYPE,“cp” ) ) AND ( LIMIT-TO ( LANGUAGE,“English” ) )

**Table 3:** Mapping of included studies to their primary research goal categories.

Main Goal Category	Publications
Monitoring	[4,7,8,13,15,16,22,23,24,25,26,27,28,29,30,31,32,33,34,35,36,37,38,39,40,41,42]
Behavior Detection	[9,10,12,43,44,45,46,47,48,49,50,51,52,53,54,55,56,57,58,59,60,61,62,63,64,65,66,67,68,69,70,71,72,73,74]
Health Assessment	[11,14,75,76,77,78,79,80,81,82,83,84,85,86,87,88,89,90,91,92,93,94,95,96,97,98,99,100,101,102,103,104,105,106,107,108]
Prediction/Forecasting	[2,109,110,111,112,113,114,115,116,117,118,119,120,121,122,123,124]
Optimization/Decision Support	[125,126,127,128,129,130,131,132]
System/IoT Development	[1,3,5,6,133,134,135]
Threat Detection	[136,137,138,139,140]

**Table 4:** Adoption of analytical methods across publication periods.

Publication Period	Total Publications	Used Analytical Methods	% with Methods
Before 2015	2	0	0.0%
2015–2018	16	11	68.8%
2019–2022	32	26	81.2%
2023–2025	85	81	95.3%

**Table 5:** Representative sensor types used in smart beehive studies, including typical accuracy and approximate cost.

Sensor Category	Example Device/Modality	Typical Accuracy	Approx. Cost *^a^*
Temperature (internal or external)	DS18B20 digital probe	±0.5 °C	$2–5 per sensor
Weight/load sensing	Four strain-gauge load cells with HX711 ADC	±0.1kg (approx. 0.02% full scale)	$20–30 for four sensors
Acoustic/vibration	Electret microphone (audio sampling for soundscape)	Frequency response 20 Hz–20 kHz; no intrinsic accuracy but sensitivity of −44dB	$5–10 per sensor
Imaging	Raspberry Pi camera V2 (8 MP) or USB webcam	1080p resolution; shutter speeds down to 30 µs	$25–35 per camera
Air composition	MQ-135 CO_2_ sensor or Figaro TGS series	±(100 ppm + 5% of reading) for CO_2_ concentration	$10–20 per sensor
Bee activity counters	Infrared gate or RFID tag	Counting accuracy 90–95% (dependent on traffic)	$15–25 per channel

*^a^* Retail prices in USD, as of 2025.

**Table 6:** Reported performance metrics for exemplar smart-beehive algorithms. Metrics correspond to the best models reported in the cited studies.

Application Task	Reported Performance
Queen absence/presence detection (microclimate or audio)	Achieved >97% accuracy using MFCC features in int16; 93% with STFT in int32 [95]; Microclimate dataset: KNN, MLP, SVM: 100% accuracy; Bioacustic dataset: MLP: 98.2% accuracy [93]; CNNs (e.g., ResNet-50) achieved up to 99% accuracy [91].
Drone vs. worker beeclassification (audio)	99.88% accuracy using Random Forest and 99.68% using KNN [53]; MUSIC + NN3 + T*: 99.97%, GTCC + NN3 + T*: 99.94%, Burg/MFCC + NN4 + T*: ≥99.85% [61]; Burg method (parametric PSC): Accuracy = 95.9%, Blackman-Tukey method: Accuracy = 94.79% [71].
Swarm prediction/weight forecasting	Best LSTM performance was achieved with a 2-h prediction window, using a 4-hour input window, where RMSE ranged from 0.042 °C to 0.217 °C across hives [113]; Vector Error Correction Model (VEC) outperformed other models in most cases, showing: 1-day ahead MAEs: Temperature: 0.6–2.4 °C, Humidity: 2.4–10.9%, Weight: 63–178 g (3-day ahead predictions remained within similar error margins.) [120]; All model types (ANN, CNN, LSTM, ARIMA) were able to predict short-term and long-term trends of thethree variables [121].
Bee counting in images	This study used a dataset of 2300 annotated images and 7200 frames, training YOLOv8 to detect bees with high accuracy and robustness under variable lighting. The best model achieved a mean Average Precision (mAP@0.5) of 0.948, an F1-score of 0.91, and precision of 1.00 at a confidence threshold of 0.838. [56]; Best pipeline: YOLOv8m + OC-SORT + Box Method, achieving F1-in = 91.49%, F1-out = 89.08%, and FPS = 21.99 [57].
Mite detection on bee images	Bee detection had F1 ≈ 0.8 and precision up to 1.0, while Varroa detection showed TPR = 0.94, TNR = 0.92, F1 ≈ 0.8, and precision ≈ 0.7. Camera resolution strongly impacted detection effectiveness—5 MP required for reliable results, [77]; The authors developed and validated a deep learning model (Faster R-CNN + ResNet-FPN backbone): mAP (mean Average Precision): 0.907, mAR (mean Average Recall): 0.967. These scores were reached using ResNet50-FPN, confidence threshold of 0.5, refinement, and DeblurGAN [87]; YOLOv5s achieved best Varroa mite detection: mAP@0.5 = 0.974, Precision = 0.962, Recall = 0.967. YOLOv5n was fastest: 4.5 ms/image [99].
Activity anomalydetection (multimodal)	Achieved 99.7% accuracy and 87% F1 score on swarm detection using AE trained on spectrograms. Pre-swarming detection was more difficult: AE reached only 60% accuracy, 22–24% F1, vs. 76.4% accuracy with RF [66]; The fuzzy logic model achieved 98% accuracy, 100% precision, 97% recall, and 98% F1-score in colony state detection. It successfully identified events like swarming, colony death, and temperature anomalies based solely on hive temperature profiles [78]; Robust regression had R^2^ ≈ 0.95–0.997, and alarms could be triggered when observed values fall outside prediction intervals [86].

**Table 7:** Summary of publicly available smart-beehive datasets by modality and ML application.

Dataset Title	Modality	Typical ML Purpose
To bee or not to bee: An annotated dataset for beehive sound recognition [141]	Acoustic	Binary sound classification (Bee vs. noBee)
Audio-Based identification of Beehive states: The dataset [142]	Acoustic	Multi-class classification of calm/pre-swarm/swarm hive states
Beehive Sounds [143]	Acoustic	State classification (healthy, distressed, empty); anomaly detection
Dataset for honey bee audio detection [18]	Acoustic	Species classification (bee vs. drone) using spectrograms
Queenless honeybee acoustic patterns [144]	Acoustic	Queen state detection
Labeled dataset for bee detection and direction estimation on beehive landingboards [145]	Visual	Object detection, pose estimation, and behavior tracking from video
Dataset for varroa mite detection on stickyboards [146]	Visual	Varroa mite detection
VarroaDataset [17]	Visual	Parasite detection (Varroa destructor); object detection with bounding boxes
VnPollenBee Dataset [147]	Visual	Pollen-bee classification
Honey Bee Annotated Images [148]	Visual	Bee detection and classification
Research project on field data collection for honey bee colony model evaluation [149]	Multimodal	Colony behavior/risk modeling; multi-source integration
Bee colony remote monitoring based on IoT using ESP-NOW protocol [37]	Environmental	Colony state monitoring using temperature, weight, battery data for predictive modeling
Winter carbon dioxide measurements in UK honeybee hives 2022/2023 [150]	Environmental	Winter vitality prediction
NASA POWER [151]	Environmental	External environmental feature augmentation for beehive activity modeling

## Data Availability

Not applicable.

## Appendix A. Detailed Summary of Included Studies

This appendix provides the full table summarizing the 135 included studies. The table lists each publication along with its deployed sensor modalities, communication technologies, and analytical methods. In the main text we present aggregated analyses and condensed tables for readability.

**Table A1:** Summary of included studies (N = 135), categorized by sensor modality, communication protocol, and analytical technique.

PUBLICATION	Sensor/Data Type	Communication Type	Method/Algorithm
Henry et al. [1]	Temperature, Humidity, Microphone	WiFi, Ethernet	–
Ochoa et al. [3]	Temperature, Humidity, Weight scale	WiFi	–
Khairul et al. [4]	Temperature, Humidity, Weight scale	WiFi	–
Zabasta et al. [5]	Temperature, Humidity, Weight scale, Camera	WiFi, GSM/GPRS, RF	–
Komasilovs et al. [6]	Temperature, Weight scale, Microphone	WiFi, GSM/GPRS	Fast Fourier Transform (FFT), Data aggregation techniques (AVG, COUNT)
Zacepins et al. [2]	Temperature	WiFi	Custom swarming algorithm
Sánchez et al. [7]	Temperature, Humidity	–	–
Li et al. [9]	Temperature, Humidity	–	ANOVA
Kale et al. [10]	Camera	–	Gaussian Mixture Models (GMM), Cascade classification, Optical flow
Gil-Lebrero et al. [8]	Temperature, Humidity, Weight scale	ZigBee	–
Kviesis et al. [11]	Temperature	–	Neural network
Rybin et al. [12]	Temperature, Humidity, Weight scale, Microphone	RF	Wavelet transformation, Neural network
Edwards-Murphy et al. [13]	Temperature, Humidity, CO_2_, O_2_, NO_2_, Pollutant levels, Accelerometer	GSM/GPRS, ZigBee	Custom temperature and humidity algorithm and CO_2_
Edwards-Murphy et al. [14]	Temperature, Humidity, CO_2_, O_2_, NO_2_, Pollutant levels, Accelerometer	GSM/GPRS, ZigBee	Decision Trees (C4.5), Custom temperature and humidity algorithm and CO_2_
Kridi et al. [15]	Temperature	ZigBee	k-means clustering
Edwards-Murphy et al. [16]	Microphone, Accelerometer, Infrared camera, Thermal camera	GSM/GPRS, ZigBee	–
Edwards-Murphy et al. [22]	Temperature, Humidity, CO_2_, O_2_, NO_2_, Pollutant levels, Accelerometer	GSM/GPRS, ZigBee	–
Žgank [43]	Microphone	WiFi, GSM/GPRS	Hidden Markov Models (HMM), Mel-Frequency Cepstral Coefficients (MFCC)
Marstaller et al. [75]	Camera	–	Neural network
Kulyukin, et al. [45]	Temperature, Microphone	–	k-means clustering, Non-Uniform Fast Fourier Transform(NFFT), Short term Fourier transform (STFT), Mel-Frequency Cepstral Coefficients (MFCC), Neural network, Support vector machine (SVM), Logistic Regression, Random Forest
Kulyukin, et al. [44]	Temperature, Microphone, Camera	–	Neural network, Support vector machine (SVM), Random Forest
Liu, et al. [23]	Temperature, Solar radiation, Wind speed and direction, Weight scale	–	–
Tu, et al. [46]	Camera	–	k-means clustering, Linear regression
Szczurek, et al. [76]	Gas sensor	–	ANOVA, Tukey’s test
STRUYE, et al. [47]	Counter	–	asynchronous sequential algorithm
Ramsey, et al. [48]	Accelerometer	–	–
Andrijević et al. [109]	Temperature, Gas sensors (TGS serise from Figaro Eng), Solar radiation, UV index, IR inensity, Rain detection, Wind speed and direction, Humidity, Microphone, Air Quality, Counter	GSM/GPRS	LSTM neural networks, Facebook Prophet, ARIMA
Voudiotis et al. [110]	Camera	LoRaWAN, WiFi	CNN
Mrozek et al. [77]	Camera	GSM/GPRS	CNN
Aydin et al. [24]	Temperature, Air Pressure, Gas sensors (TGS serise from Figaro Eng), Humidity, Weight scale	WiFi, ZigBee	–
Robustillo et al. [111]	Temperature, Air Pressure, Solar radiation, Rain detection, Wind speed and direction, Humidity, Pollutant levels	–	Vector Autoregressive (VAR), Dynamic Linear Model (DLM), Generalized Additive Model (GAM)
Hong et al. [25]	Temperature, Humidity, Weight scale, Microphone, Counter	WiFi	–
Kviesis et al. [78]	Temperature	–	fuzzy logic model (FLM)
Braga et al. [79]	Temperature, Dew point, Solar radiation, Rain detection, Wind speed and direction, Weight scale	Bluetooth	Neural network, Random Forest, k-nearest neighbors (KNN)
Li et al. [80]	Temperature, Humidity, Weight scale, Microphone, Counter	WiFi, GSM/GPRS	Data correlation
Imoize et al. [26]	Temperature, Microphone	WiFi, GSM/GPRS	Signal patterns
Cecchi et al. [27]	Temperature, Humidity, CO2, Weight scale, Microphone	WiFi, Ethernet	Signal patterns
Kaplan et al. [81]	Camera	–	VGG19, GoogLeNet
Zacepins et al. [28]	Temperature, Humidity, Weight scale	WiFi	event detection via thresholds and time-interval-based rules
Bermig et al. [49]	Temperature, Humidity, Camera, Counter	–	Manual video inspection and Robber’s test
Braga et al. [112]	Temperature, Humidity, Weight scale	–	k-means clustering, Random Forest, k-nearest neighbors (KNN)
Alves et al. [82]	Camera	–	CNNs (MobileNet, DenseNet, Inception, ResNet, etc.), U-Net for segmentation, CHT for detection, Naive Bayes (NB)
Ngo et al. [50]	Temperature, Light illuminance, Rain detection, Wind speed and direction, Humidity, Camera	WiFi	Yolov3-tiny, Majority voting, Object tracking
Sevin et al. [83]	Camera	WiFi	Shape and color-based image filtering (bee and mite templates), 3-stage detection process
Kim et al. [84]	Microphone	–	Mel-Frequency Cepstral Coefficients (MFCC), Support vector machine (SVM), Random Forest, XGBoost (gradient boosting), VGG19, Shallow CNN, Grad- CAM, CQT (Constant Q transform)
Catania et al. [29]	Temperature, Wind speed and direction, Humidity, Weight scale	Bluetooth	statistical correlation + environmental trend analysis
Braga et al. [113]	Temperature, Humidity, Weight scale, Microphone	–	LSTM neural networks, AdamX optimizer
Schurischuster et al. [85]	Camera	–	AlexNet, ResNet, Deeplabv3 (semantic segmentation)
Williams, et al. [51]	Camera, Thermal camera	–	Gaussian Mixture Models (GMM), Neural network, Support vector machine (SVM), Random Forest, k-nearest neighbors (KNN)
Žgank [52]	Microphone	WiFi, GSM/GPRS	Hidden Markov Models (HMM), Gaussian Mixture Models (GMM), Linear Predictive Coding (LPC), Mel-Frequency Cepstral Coefficients (MFCC)
Rodias et al. [125]	Temperature, Humidity, GPS module, LIDAR	–	BFCI formula: θ·T+b·P+c·W (weather scoring);
Libal et al. [53]	Microphone	–	Mel-Frequency Cepstral Coefficients (MFCC), Support vector machine (SVM), Linear Discriminant Analysis (LDA), Random Forest, k-nearest neighbors (KNN)
Chien et al. [136]	Camera	WiFi	YOLOv7
Penaloza-Aponte et al. [54]	Tags, Camera	WiFi	–
Degenfellner et al. [86]	Weight scale, Enviromental data	GSM/GPRS	Facebook Prophet, Similar Trend Monitoring (STM), Similar Trend Monitoring (STM).1, Principal Component Analysis (PCA), MM-Regression
Kongsilp et al. [55]	Camera	–	Kalman filter, Hungarian algorithm, Mask R-CNN
Divasón et al. [87]	Camera	–	Faster R-CNN with ResNet18/50/152 + FPN backbones, DeblurGAN
Chowdhury et al. [56]	Camera	–	YOLOv8
Libal et al. [114]	Microphone	–	Mel-Frequency Cepstral Coefficients (MFCC), LASSO regression, Autoencoder neural networks
Bairo et al. [30]	Weight scale	GSM/GPRS	Custom calibration model using linear regression on resistance-voltage-weight relationship
Camayo et al. [88]	Temperature, Humidity, CO2, TVOC	WiFi	Neural network, Random Forest, Decision Trees (C4.5), Weighted multi-criteria aggregation algorithm, Data aggregation techniques (AVG, COUNT), XGBoost (gradient boosting)
Liyanage et al. [126]	Temperature, Rain detection, Humidity, Air Quality	WiFi	event detection via thresholds and time-interval-based rules
Narcia-Macias et al. [89]	Temperature, Humidity, Camera	–	YOLOv7
Minaud et al. [115]	Temperature	–	Generalized Additive Model (GAM), event detection via thresholds and time-interval-based rules, RP_median thermal index, GLM validations
Garcao et al. [90]	Temperature, Humidity, Microphone	WiFi	CNN, Logistic Regression, k-nearest neighbors (KNN), Principal Component Analysis (PCA), YAMNET, VGGish, Feedforward neural network (FNN), Kendall’s tau
Pérez-Delgado et al. [140]	Camera	–	CNN
Kamga et al. [116]	Enviromental data, Local land cover quality index (LLCQI),	–	ANFIS-SC (Adaptive Neuro-Fuzzy Inference System + Subtractive Clustering)
Kontogiannis et al. [91]	Temperature, Humidity, Microphone	WiFi	CNNs (VGG-16/19, ResNet-18/50, WideResNet, Inception), Fuzzy-stranded-NN
Smerkol et al. [127]	Temperature, Air Pressure, Rain detection, Humidity, Weight scale	NBIoT	Support vector machine (SVM), Random Forest, Decision Trees (C4.5), ADABOOST, Gradient Boost
Lei et al. [57]	Camera	–	YOLOv8m, OC-SORT, BOX-METHOD
Nguyen et al. [58]	Camera	–	CNN, YOLOv5, Faster RCNN, Focal Loss, Overlap Sampler
Robles-Guerrero et al. [92]	Microphone	–	CNNs: EfficientNet, ConvNeXt, MobileNet, ShuffleNet, ResNet18, etc.
Hall et al. [137]	Microphone, Camera	–	Principal Component Analysis (PCA), Discriminant Function Analysis (DFA), 2D Fourier Transform (2DFT), classification via DF-space projection
Bono et al. [117]	Temperature, UV index, Rain detection, Wind speed and direction, Humidity, Weight scale, Microphone	GSM/GPRS	Vector Autoregressive (VAR), impulse response functions (IRF), Granger causality tests
Ramirez-Diaz et al. [118]	Enviromental data	–	Random Forest, Decision Trees (C4.5), XGBoost (gradient boosting), Boruta FS
Ho et al. [59]	Microphone	–	Fast Fourier Transform (FFT), Short term Fourier transform (STFT), Mel-Frequency Cepstral Coefficients (MFCC), Support vector machine (SVM), Logistic Regression, Random Forest, Extra Trees (ET), k-nearest neighbors (KNN), CQT (Constant Q transform), Spectral Contrast
Várkonyi et al. [119]	Microphone	–	Short term Fourier transform (STFT), Mel-Frequency Cepstral Coefficients (MFCC), Spectral Centroid, Zero Crossing Rate, Histogram-based Gradient Boosting + GA-based feature selection (regression)
Karan et al. [31]	Temperature, Light illuminance, Humidity, Weight scale, Microphone, Accelerometer	WiFi	event detection via thresholds and time-interval-based rules
Lee et al. [133]	Temperature, Humidity, CO_2_, O_2_, Weight scale, Counter	IR, power line communication (PLC)	–
Robustillo et al. [120]	Temperature, Air Pressure, Light illuminance, Rain detection, Wind speed and direction, Humidity, particulate matter, Weight scale	–	Vector Autoregressive (VAR), Dynamic Factor Analysis (DFA), ombining data from multiple time series (CMTS), eneral multivariate auto-regressive state-space (MARSG), Vector Error Correction (VEC)
Sledevič et al. [64]	Camera	–	YOLOv8-pose (nano, medium, large)
Otesbelgue et al. [93]	Temperature, Humidity, Microphone	–	Support vector machine (SVM), Random Forest, k-nearest neighbors (KNN), multilayer perceptron (MLP), extreme learning machine (ELM)
Libal et al. [61]	Microphone	–	Mel-Frequency Cepstral Coefficients (MFCC), gammatone cepstral coefficients (GTCC), BURG algorithm, MUltiple SIgnal Classification (MUSIC), Autoencoder, thresholding (T1, T2, T3, T*), empirical Bayes classifier (ML thresholding)
Kulyukin et al. [121]	Temperature, Weight scale, Camera	–	ANN, CNN, LSTM neural networks, ARIMA
Micheli et al. [62]	Camera	–	Gaussian derivative (GDER), Gray-level local variance (GLLV), Steerable filters (SFIL), Tenengrad (TENG), and Tenengrad variance (TENV), t-distributed Stochastic Neighbor Embedding (t-SNE)
Dickson et al. [63]	Camera	–	Kalman filter, YOLOv8, Optical Flow + polynomial regression
Gaikwad et al. [32]	Temperature, Humidity, Weight scale	–	event detection via thresholds and time-interval-based rules
Sledevič et al. [60]	Camera	–	YOLOv8m + YOLOv8n-seg for detection & direction, rule-based behavior detection for 4 patterns (foraging, fanning, defense, washboarding), BoT-SORT, ByteTrack, StrongSORT, DeepOC-SORT, OC-SORT tracking algorithms
Luz et al. [94]	Microphone	–	Mel-Frequency Cepstral Coefficients (MFCC), Support vector machine (SVM), Random Forest, multilayer perceptron (MLP), VGG16/ResNet50/MobileNet/YOLO, Mel spectrograms
Iqbal et al. [65]	Microphone	–	Mel-Frequency Cepstral Coefficients (MFCC), CNN, LSTM neural networks, Support vector machine (SVM), k-nearest neighbors (KNN), Naive Bayes (NB), Mel spectrograms, transformer mode
De Simone et al. [95]	Microphone	LoRaWAN	Short term Fourier transform (STFT), Mel-Frequency Cepstral Coefficients (MFCC), TinyML neural network (3-layer NN)
Newton et al. [96]	Temperature, Humidity, CO_2_, Weight scale, Vibration	–	analysis based on signal tracking, vibration spectrograms, and time-series trends
Zheng et al. [33]	Temperature, Humidity, Microphone, Camera	WiFi	YOLOv5, DeepSORT, rule-based bee entry/exit/count logic
Alifieris et al. [134]	Temperature, Air Pressure, Humidity, Weight scale, Enviromental data, Microphone	LoRaWAN, WiFi, GSM/GPRS	rule-based journaling, checklist mapping, data stream aggregation
Janetzky et al. [66]	Microphone	–	Random Forest, Isolation Forrest, Principal Component Analysis (PCA), Autoencoder neural networks, Spectrograms
Rathore et al. [97]	Camera	–	CLAHE (contrast enhancement), Bilateral filter, Hough Circle Transform
Borgianni et al. [98]	Microphone	–	DenseNet121, ResNet50, InceptionV3, VGG16; Federated Averaging (FedAvg), CNN-based DNNs (spectrogram input)
Kulyukin et al. [122]	Electromgnetic radiation (EMR), Air Pressure, Solar radiation, Rain detection, Wind speed and direction, Humidity, Camera	–	Support vector machine (SVM), Linear regression, Random Forest
Várkonyi et al. [67]	Microphone	–	Short term Fourier transform (STFT), Mel-Frequency Cepstral Coefficients (MFCC), MFCC differential coefficients (MFCC delta), CNN, LSTM neural networks, Spectral Centroid, Zero Crossing Rate, DANi NF method, Chroma
Williams et al. [68]	Camera, Doppler radar counter	–	Linear Predictive Coding (LPC), Support vector machine (SVM), DenseNet, Log Area Ratios (LAR)
Mahajan et al. [99]	Microphone, Camera	–	Mel-Frequency Cepstral Coefficients (MFCC), YOLOv7, YOLOv8, Mel spectrograms, Single Shot Multibox Detector (SSD), Detection Transformer (DETR), Dense NN (2-layer MLP on MFCC+Mel features)
Cota et al. [34]	Temperature, Lid microswitch, Humidity, Weight scale, Microphone, GPS module	WiFi	event detection via thresholds and time-interval-based rules
Vallone et al. [132]	Temperature, Humidity, Weight scale, Microphone	GSM/GPRS	Rule-based trend evaluation for honey production and swarm behavior prediction
Abdollahi et al. [35]	Microphone	–	Short-Time Energy, WebRTC VAD, CRDNN
Vit et al. [128]	Camera	–	CNNs (VGG19, DenseNet121, EfficientNetV2S, ResNet50, InceptionV3)
Kulyukin et al. [69]	Camera	WiFi	YOLOv3, YOLOv4-tiny, YOLOv7-tiny, OmniBeeM
Jeon et al. [138]	Camera	GSM/GPRS	YOLOv5s
Wu et al. [123]	Tags, Dew point, Air Pressure, Solar radiation, UV index, Rain detection, Wind speed and direction, Humidity	–	LSTM neural networks, gated recurrent unit (GRU)
Safie et al. [70]	Camera	–	YOLOv3, SqueezeNet (18-layer CNN), DarkNet-53 (53-layer CNN)
Milovanovic et al. [36]	64 IR opto-reflective sensors	WiFi	–
Phan et al. [129]	Microphone	–	Logistic Regression, Random Forest, Decision Trees (C4.5), Extra Trees (ET), XGBoost (gradient boosting), k-nearest neighbors (KNN)
Kviesis et al. [37]	Temperature, Weight scale	GSM/GPRS	event detection via thresholds and time-interval-based rules, CNN-based DNNs (spectrogram input)
Abdollahi et al. [100]	Temperature, Humidity, Microphone	–	discrete wavelet transform (DWT), Mel-Frequency Cepstral Coefficients (MFCC), Spectrograms
Campell et al. [124]	Temperature, Humidity, Weight scale, Microphone, Camera	–	Short term Fourier transform (STFT), Non-Negative Matrix Factorization (NMF), Masked NMF, Minimum Covariance Determinant (MCD), ANN
Grammalidis et al. [130]	Temperature, Humidity, Camera, Microscope images, Satellite images	–	CNN, Mask R-CNN, U-TAE (Transformer + U-Net), YOLOv6
Florea et al. [131]	Temperature, Air Pressure, Humidity, Microphone, ultrasonic distance	–	event detection via thresholds and time-interval-based rules
Libal et al. [71]	Microphone	–	Fast Fourier Transform (FFT), BURG algorithm, Autoencoder neural networks, Blackman-Tukey
Chen et al. [38]	Temperature, Humidity, Weight scale, Counter	LoRaWAN	event detection via thresholds and time-interval-based rules
Sledevič et al. [72]	Camera	–	YOLOv8m
Sharma et al. [101]	Camera	–	CLAHE (contrast enhancement), CNN (ResNet-50, Inception V3)
Sledevič et al. [73]	Camera	–	YOLOv8m
Barbisan et al. [102]	Microphone	–	Short term Fourier transform (STFT), Mel-Frequency Cepstral Coefficients (MFCC), Support vector machine (SVM), multilayer perceptron (MLP)
Durga et al. [103]	Camera	–	Vision Transformer (ViT14, ViT16, ViT32)
De Simone et al. [104]	Microphone	–	Short term Fourier transform (STFT), Mel-Frequency Cepstral Coefficients (MFCC), 2-layer NN
Hamza, et al. [39]	Temperature, Humidity, Weight scale, Microphone	–	event detection via thresholds and time-interval-based rules
Sanz, et al. [40]	Temperature, Air Pressure, Humidity	LoRaWAN	Statistical analysis (ANOVA + Fisher LSD)
Ruvinga, et al. [105]	Microphone	–	Short term Fourier transform (STFT), Mel-Frequency Cepstral Coefficients (MFCC), CNN, LSTM neural networks, Logistic Regression, multilayer perceptron (MLP)
Thi, et al. [74]	Microphone	–	Genetic Programming (GP)
Lee, et al. [106]	Camera	–	ORB (Oriented FAST and Rotated BRIEF), Contrast-Limited Adaptive Histogram Equalization (CLAHE), RGB/HSV/Lab/Gray/YCrCb color models, Histogram Equalization
Capela, et al. [41]	Weight scale, Camera	–	DeepBee# (custom-trained CNN)
Dokukin, et al. [107]	Microphone	–	Support vector machine (SVM), Logistic Regression, Random Forest, XGBoost (gradient boosting), Statistically Weighted Syndrome (SWS), OVP method
Nasir et al. [139]	Camera, Infrared camera	–	Xception, GoogLeNet, Ensemble Bagged Trees, Multi-evidence fusion via weighted voting
Milovanović et al. [42]	900 IR photo reflectors	GSM/GPRS	Reflectivity-based classification (voltage thresholds), Absorption spectroscopy
Divasón et al. [108]	Camera	–	Faster R-CNN + ResNet50-FPN backbone, Enhanced Deep Super-Resolution (EDSR), Stochasticgradientdescent(SGD)
Braga et al. [135]	Vibration, GPS module	GSM/GPRS	Rule-based detection: vibration triggers + GPS tracking + notification logic
